# c-Myc inhibition and p21 modulation contribute to unsymmetrical bisacridines-induced apoptosis and senescence in pancreatic cancer cells

**DOI:** 10.1007/s43440-024-00658-6

**Published:** 2024-10-03

**Authors:** Agnieszka Kurdyn, Monika Pawłowska, Ewa Paluszkiewicz, Mirosława Cichorek, Ewa Augustin

**Affiliations:** 1 Department of Pharmaceutical Technology and Biochemistry, Faculty of Chemistry, Gdańsk University of Technology, Gabriela Narutowicza 11/12, Gdańsk, 80-233 Poland; 2 Department of Embryology, Medical University of Gdańsk, Dębinki 1, Gdańsk, 80-211 Poland

**Keywords:** Unsymmetrical bisacridines, Pancreatic cancer, Apoptosis, Senescence, Anticancer activity

## Abstract

**Background:**

Pancreatic cancer (PC) is one of the most aggressive cancers and is the seventh leading cause of cancer-related death worldwide. PC is characterized by rapid progression and resistance to conventional treatments. Mutations in *KRAS*,* CDKN2A*, *TP53*,* SMAD4/DPC4*, and *MYC* are major genetic alterations associated with poor treatment outcomes in patients with PC. Therefore, optimizing PC therapy is a tremendous challenge. Unsymmetrical bisacridines (UAs), synthesized by our group, are new promising compounds that have exhibited high cytotoxicity and antitumor activity against several solid tumors, including pancreatic cancer.

**Methods:**

The cellular effects induced by UAs in PC cells were evaluated by MTT assay (cell growth inhibition), flow cytometry, and fluorescence and light microscopy (cell cycle distribution, apoptosis, and senescence detection). Analysis of the effects of UAs on the levels of proteins (c-Myc, p53, SMAD4, p21, and p16) was performed by Western blotting.

**Results:**

Apoptosis was the main triggered mechanism of death after UAs treatment, and induction of the SMAD4 protein can facilitate this process. c-Myc, which is one of the molecular targets of UAs, can participate in the induction of cell death in a p53-independent manner. Moreover, UAs can also induce accelerated senescence through the upregulation of p21. Notably, senescent cells can die via apoptosis after prolonged exposure to UAs.

**Conclusions:**

UAs have emerged as potent anticancer agents that induce apoptosis by inhibiting c-Myc protein and triggering cellular senescence in a dose-dependent manner by increasing p21 levels. Thus, UAs exhibit desirable features as promising candidates for future pancreatic anticancer therapies.

**Supplementary Information:**

The online version contains supplementary material available at 10.1007/s43440-024-00658-6.

## Introduction

Pancreatic cancer (PC) is a highly lethal cancer and is the seventh most common cause of cancer-related death worldwide. It is estimated that PC will become the second leading cause of tumor-related mortality in the next 10 years [1]. This negative prognosis results from its late diagnosis, lack of early diagnostic biomarkers [2], and great genetic, intra- and intertumoral heterogeneity compared with other solid tumors [3]. Moreover, the accumulation of genetic alterations in pancreatic cells leads to multistage transformation and, consequently, malignancy, which is often diagnosed at an advanced stage due to the lack of early symptoms [4]. Key mutations detected in PC involve the proto-oncogene *KRAS* and the tumor suppressors *CDKN2A*, *TP53*, and *SMAD4/DPC4*, which are associated with cell cycle deregulation, apoptosis inhibition, invasion, metastasis, and poor treatment outcomes [5]. In addition, the c-Myc protein is commonly overexpressed in pancreatic cancer, enhancing proliferation, invasion, metastasis, angiogenesis, and evasion of the immune response [6, 7].

*KRAS* mutations are most common in PC, with more than 90% of tumors harboring oncogenic point mutations in this gene. Mutant K-Ras initiates a cascade that provides a strong pro-growth signal, increases cell motility and invasion, and profoundly rearranges cell metabolism to a growth-promoting state. One of the downstream proteins of K-Ras is the transcription factor, c-Myc, which is estimated to regulate 15% of genes in humans [8] and can act as a node in integrating several important pathways that are mutated in pancreatic cancer [9].

Because the inhibition of c-Myc attenuates chemoresistance and enhances immunotherapy, there is a high demand for compounds that directly or indirectly target this protein [6]. c-Myc has been shown to inhibit the transcription of p21, an important regulator of cell cycle control checkpoints, through its interaction and subsequent repression of multiple transcription factors [10]. Furthermore, c-Myc shares a binding domain on p21 with proliferating cell nuclear antigen (PCNA) and thereby can disrupt the interaction of p21 with PCNA, consequently reducing the inhibition of DNA synthesis [11]. Therefore, the protective function of p21 is disabled in tumor cells such as those in pancreatic cancer with high c-Myc levels [12]. In addition to its roles in cell cycle regulation, p21 is involved in differentiation, cell migration, cytoskeletal dynamics, apoptosis, transcription, DNA repair, reprogramming of induced pluripotent stem cells, autophagy, and the onset of senescence. The p21 protein is transcriptionally controlled by both p53-dependent and p53-independent pathways [10]. Inactivating mutations in the *TP53* gene have been reported in more than 50% of pancreatic cancers and commonly result in apoptosis evasion, loss of cell cycle control, and the disabling of DNA damage repair [13]. *TP53* is typically altered through gain-of-function missense mutations that may further promote cancer beyond the loss of classical functions of this tumor suppressor [14].

Another commonly mutated gene in pancreatic cancer is *CDKN2A* (> 40%), whose alterations are related mainly to cell cycle dysregulation, promoting its progression through the loss of cyclin-dependent kinase CDK4 and CDK6 inhibition [15]. In turn, the gene encoding the tumor suppressor protein SMAD4 is inactivated in 50–55% of pancreatic cancers, and the role of its loss in PC remains controversial but appears to be associated with poorer overall survival and metastasis. SMAD4 is the central signal transducer of the transforming growth factor-β (TGF-β) pathway, which is known to play a role in epithelial‒mesenchymal transition (EMT) [16].

According to the latest studies, the standard of care for pancreatic cancer has been gemcitabine, nab-paclitaxel, and the extremely toxic FOLFIRINOX (folinic acid, fluorouracil, irinotecan, and oxaliplatin). However, current chemotherapy approaches for treating PC are still minimally effective in terms of survival rates [17–19]. Considering the poor prognosis of patients with pancreatic cancer and the limited achievements in its treatment, new chemotherapeutic agents are needed to improve its management [6].

**Fig. 1:**
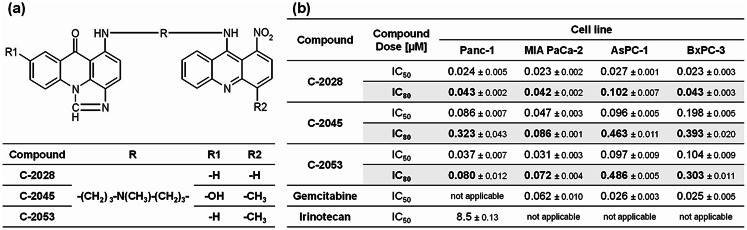
Chemical structures and IC (inhibitory concentrations) doses of UA compounds. (**a**) The chemical structures of UA derivatives: C-2028, C-2045, and C-2053. (**b**) IC doses of UAs and positive controls (gemcitabine or irinotecan) against Panc-1, MIA PaCa-2, AsPC-1, and BxPC-3 cells after 72 h of treatment. R, R1, R2– substituents. Data are expressed as IC_50_ and IC_80_ (mean ± SD, *n* ≥ 4)

Unsymmetrical bisacridines (UAs) (Fig. 1a) are a new group of antitumor compounds among acridine derivatives synthesized at Gdańsk University of Technology. These compounds are patented in Europe [20], the USA [21], Japan [22], and Canada [23] and exhibit high cytotoxicity and antitumor activity toward human tumor xenografts in nude mice, preferentially against human pancreatic cancer and colon, lung, prostate, and breast cancers [24]. We have previously shown that UAs interact with several DNA G-quadruplexes, especially with those present in the promoters of the *KRAS* and *MYC* oncogenes [25]. Additionally, our previous study revealed that UAs affect c-Myc protein levels in a manner dependent on the type of cells treated. A reduction in the c-Myc protein level and the induction of apoptosis after UAs treatment occurred to a greater extent in H460 lung cancer cells than in HCT116 colon cancer cells [26]. Notably, DNA G-quadruplexes are present in the telomeric and promoter regions of oncogenes, which are frequently mutated in various types of cancer and encode the following proteins: c-Myc, Bcl-2, PDFGR-β, VEGR, K-Ras, and Kit [27, 28]. Molecules that can stabilize the DNA G-quadruplex have been shown to exert antiproliferative and chemosensitizing effects in in vitro and in vivo tumor models without appreciably affecting normal cells.

Considering that UAs show different activities depending on the type of treated cells [26] and that preliminary in vitro and in vivo studies have shown the high cytotoxic and antitumor activity of UAs against pancreatic cancer cells [24], the present study aimed to investigate the cellular mechanism of action of the selected UA derivatives in pancreatic cancer cells, with a particular focus on the type of induced cell death and the senescence process. To more accurately represent the variety of the patient population, four pancreatic cancer cell lines with different genetic profiles, namely, Panc-1, MIA PaCa-2, AsPC-1, and BxPC-3, were selected for this study. In addition, to identify the roles of proteins that may be involved in the cellular response induced by UAs, we intend to examine proteins such as c-Myc, p53, p21, and SMAD4, which are frequently mutated in pancreatic cancer or have G-quadruplexes in their promoter regions.

## Materials and methods

### Materials

2-Mercaptoethanol (Sigma-Aldrich, St Louis, MO, USA, product cat. no. M3148), 5-Fluorouracil (Sigma-Aldrich, St Louis, MO, USA, product cat. no. F6627), Acetic acid 99.5–99.9% (POCH, Gliwice, Poland, product cat. no. 568760114), Acrylamide/Bis-acrylamide, 30% solution (Sigma-Aldrich, St Louis, MO, USA, product cat. no. A3699), Ammonium persulfate (APS, Sigma-Aldrich, St Louis, MO, USA, product cat. no. A3678), Cisplatin (Sigma-Aldrich, St Louis, MO, USA, product cat. no. PHR1624), Citric acid monohydrate pure (POCH, Gliwice, Poland, product cat. no. 538210118), Erlotinib (Cayman Chemical Company, Ann Arbor, MI, USA, product cat. no. 10483) Ethyl alcohol 96% (POCH, Gliwice, Poland, product cat. no. 396420113), Formaldehyde 36–38% (POCH, Gliwice, Poland, product cat. no. 432173111), Gemcitabine hydrochloride (Sigma-Aldrich, St Louis, MO, USA, product cat. no. G6423), Glutaraldehyde solution 25% (POCH, Gliwice, Poland, product cat. no. 461023423), Glycine (Sigma-Aldrich, St Louis, MO, USA, product cat. no. G8898), Irinotecan hydrochloride (Sigma-Aldrich, St Louis, MO, USA, product cat. no. I1406), L-glutamine solution (Sigma-Aldrich, St Louis, MO, USA, product cat. no. G7513), Magnesium chloride (Sigma-Aldrich, St Louis, MO, USA, product cat. no. M8266), Methanol (POCH, Gliwice, Poland, product cat. no. 621990110), N, N-Dimethyloformamide (POCH, Gliwice, Poland, product cat. no. 355120112), N, N,N′,N′-Tetramethylethylenediamine (TEMED, Sigma-Aldrich, St Louis, MO, USA, product cat. no. T7024), Phosphate buffered saline (PBS, Sigma-Aldrich, St Louis, MO, USA, product cat. no. P4417), Potasium hexacyanoferrate (II) trihydrate (Sigma-Aldrich, St Louis, MO, USA, product cat. no. P9387), Potasium hexacyanoferrate (III) 99% (Sigma-Aldrich, St Louis, MO, USA, product cat. no. P8131), Sodium bicarbonate (Sigma-Aldrich, St Louis, MO, USA, product cat. no. S5761), Sodium chloride 99.9% (POCH, Gliwice, Poland, product cat. no. 363550117), Trizma base (Sigma-Aldrich, St Louis, MO, USA, product cat. no. T1503), Trypsin-EDTA 10× (Biowest, Riverside, MO, USA, product cat. no. X0930), Tween 20 (Sigma-Aldrich, St Louis, MO, USA, product cat. no. P9416).

### Methods

#### Cell lines and culture

In the present study the following cell lines were used: Four human pancreatic cancer cell lines (Panc-1, MIA PaCa-2, AsPC-1, BxPC-3) and one normal pancreatic cell line (hTERT-HPNE). They were purchased from the American Type Culture Collection (Manassas, VA, ATCC). Panc-1 and MIA PaCa-2 cells were maintained in high-glucose Dulbecco’s modified Eagle’s medium (DMEM HG, Sigma-Aldrich, St Louis, MO, USA, product cat. no. D5648), whereas AsPC-1 and BxPC-3 cells were maintained in RPMI 1640 medium (Sigma-Aldrich, St Louis, MO, USA, product cat. no. R0883). Both media were supplemented with heat-inactivated 10% fetal bovine serum (FBS; Biowest, Riverside, MO, USA, product cat. no. S1520), 100 µg/mL streptomycin (Sigma-Aldrich, St Louis, MO, USA, product cat. no.S9137), and 100 units/mL penicillin (Sigma-Aldrich, St Louis, MO, USA, product cat. no. P3032). hTERT-HPNE cells were maintained in 75% Dulbecco’s modified Eagle’s medium without glucose (DMEM, Sigma-Aldrich, St Louis, MO, USA, product cat. no. D5030), 25% Essential 8™ Basal Medium (Gibco, Thermo Fisher, Waltham, MA, USA, product cat. no. A1517001) supplemented with heat-inactivated 5% fetal bovine serum (FBS; Biowest, Riverside, MO, USA, product cat. no. S1520), 10ng/mL hEGF (Sigma-Aldrich, St Louis, MO, USA, product cat. no. E9644), 1 g/L D-glucose (POCH, Gliwice, Poland, product cat. no. 459560117) and 1% Penicillin-Streptomycin (Sigma-Aldrich, St Louis, MO, USA, product cat. no. P0781). Cells were incubated in a humidified atmosphere of 5% CO_2_ at 37 °C. All experiments were performed on cells in a logarithmic growth phase.

#### Cell viability assay

To investigate the effect of UAs on cell viability, a colorimetric analysis was performed using MTT (3-(4,5-Dimethyl-2-thiazolyl)-2,5-diphenyl-2 H-tetrazolium bromide, Sigma-Aldrich, St Louis, MO, USA, product cat. no. M2128) based on the reduction of this yellow salt in metabolically active cells to purple formazan crystals. Cells were seeded onto 24-well plates at 2.5 × 10^4^ cells/well for Panc-1 and BxPC-3, 2 × 10^4^ cells/well for MIA PaCa-2 and AsPC-1, and 6.5 × 10^4^ for hTERT-HPNE. After 24 h incubation for cell attachment, cells were treated with concentrations of UAs and gemcitabine up to 5 µM and irinotecan up to 100 µM. Gemcitabine was selected as the reference compound for MIA PaCa-2, AsPC-1, and BxPC-3 cells, while irinotecan for Panc-1 cells. Stock solutions (10 mM) of UAs and gemcitabine were prepared in deionized, sterile water while irinotecan was prepared in DMSO. Dilutions of all compounds were prepared in deionized, sterile water. After 72 h of incubation, 200 µL MTT at a concentration of 4 mg/mL was added to each well for 3–4 h. The culture medium was then aspirated and formazan crystals were dissolved in DMSO. The absorbance of the solutions was measured at 540 nm using an iMark Microplate Absorbance Reader (Bio-Rad, Hercules, CA, USA). The drug concentrations required to inhibit cell growth by 50 (IC_50_) and 80% (IC_80_) compared to untreated control cells were determined from curves plotting survival as a function of dose. Results were obtained from at least four independent experiments. The experiment was performed at least four times.

#### Cell cycle analysis

Cell cycle distribution was assessed by DNA content, which was determined by flow cytometry using propidium iodide (PI), a fluorescent dye that binds stoichiometrically to DNA. Cells were seeded at a density of 1.5 × 10^6^ for Panc-1, 1.2 × 10^6^ for BxPC-3, and 1 × 10^6^ for MIA PaCa-2 and AsPC-1 per 100 mm Petri dish. Cells were then treated with IC_80_ doses of UAs and IC_50_ doses of reference compounds for 24, 72, 120, and 168 h. Following exposure to the test compounds, cells were washed with cold 1X PBS buffer, trypsinized, washed twice with cold 1X PBS, and fixed in 80% (v/v) ethanol at– 20 °C for at least 24 h. Afterward, the fixed cells were washed twice with 1X PBS, stained with PI/RNase Staining Buffer (BD Biosciences, San Jose, CA, USA, product cat. no. 550,825), and analyzed using a FACS Accuri C6 flow cytometer (BD Biosciences, San Jose, CA, USA). At least 10,000 cells were collected and the results were analyzed using BD Accuri C6 Software (Version 1.0264.21). The experiment was performed at least four times.

#### Cell death determination by annexin V/propidium iodide (PI) analysis

The type of death induced by the test compounds was assessed using PI/annexin-V-FITC double staining, allowing the identification of cells with externalization of phosphatidylserine, a hallmark of apoptotic cells. Panc-1, MIA PaCa-2, AsPC-1, and BxPC-3 cells were seeded and treated with test compounds analogous to cell cycle analysis. After exposure to the compounds, cells were harvested, washed twice with cold 1X PBS, pelleted, and resuspended in 50 µL of binding buffer containing PI and annexin-V-FITC (FITC Annexin V Apoptosis Detection Kit I, BD Biosciences, San Jose, CA, USA, product cat. no. 556,547). The cells were then incubated in the dark for 15 min at room temperature. After incubation, 180 µL of binding buffer was added and samples were analyzed with a FACS Accuri C6 flow cytometer (BD Biosciences, San Jose, CA, USA). At least 10,000 cells were collected and the results were analyzed using BD Accuri C6 Software (Version 1.0264.21). The experiment was performed at least four times.

#### Assessment of cell nuclei morphology

To determine the morphology of the cell nuclei of cells treated with the test compounds, staining of the fixed cells with Hoechst 33,342 (Sigma-Aldrich, St Louis, MO, USA product cat. no. B2261), the fluorescent dye was used. This allowed analysis of chromatin condensation and fragmentation, as well as the size and multiplicity of cell nuclei. Panc-1, MIA PaCa-2, AsPC-1, and BxPC-3 cells were seeded and treated with test compounds analogous to cell cycle analysis. Successively, cells were washed with 1X PBS, resuspended in 1X PBS, and centrifuged onto slides. The cells were then fixed with methanol: acetic acid (3:1) for 15 min and stained with Hoechst 33,342 (1 mg/mL) for 15 min. The morphology of the nuclei was examined under an OLYMPUS BX60 microscope connected to an U-RFL-T lamp coupled via XC50 CCD camera to a personal computer equipped with cellSens Standard 1.18 software (Olympus, Tokyo, Japan). Images were taken using the UV filter and 400× magnification. For each sample slide, 3–4 pictures were taken that showed at least 50 cell nuclei. Cells were identified as apoptotic based on the presence of condensed, fragmented chromatin. Enlarged cells containing multiple nuclei were considered as mitotic catastrophe. Abnormal distribution of genetic material was considered as abnormal mitosis. The experiment was performed at least three times.

#### Cellular senescence observation

To determine the morphology characteristic of senescent cells and the expression of senescence-associated β-galactosidase (SA-β-Gal), a marker of this process, microscopic observation was performed. Panc-1, MIA PaCa-2, AsPC-1, and BxPC-3 cells were seeded, in a quantity that prevents the cells from reaching confluence, in 60 mm Petri dishes containing microscope coverslips and treated with UAs (IC_80_ and IC_50_ doses) and reference compounds (IC_50_ dose) for 72, 120, and 168 h. After compound treatment cells were washed twice with 1X PBS and fixed with 2% glutaraldehyde and 0.2% formaldehyde for 5 min. Next cells were washed twice with 1X PBS (pH 6.0) and coverslips were moved to 35 mm Petri dishes and incubated at pH 6.0 with X-gal (5-bromo-4-chloro-3-indolyl-β-D-galactosidase, A&A Biotechnology, Poland, product cat. no. B4252) staining solution (1 mg/mL Χ-Gal, 40 mmol/L citric acid/sodium phosphate, pH 6.0, 5 mmol/L potassium hexacyanoferrate(II), 5 mmol/L potassium hexacyanoferrate(III), 150 mmol/L NaCl, 2 mmol/L MgCl_2_). After overnight incubation at 37 °C, cells were washed twice with 1X PBS pH 7.2 and observed under an OLYMPUS BX60 microscope coupled via XC50 CCD camera to a personal computer equipped with a cellSens Standard 1.18 software (Olympus, Tokyo, Japan). Images were taken in light mode using the Nomarski interference contrast at 200× magnification. At least 5 images were taken for each slide, a minimum of 400 cells were counted and those stained blue were identified as senescent cells. The experiment was performed at least two times.

#### Cytofluorimetric analysis of SA-β-Gal activity

The CellEvent™ Senescence Green Flow Cytometry Assay Kit (Invitrogen, Thermo Fisher, Waltham, MA, USA, product cat. no. C10841) is a fluorescent-based reagent that contains two galactoside moieties (C12FDG; 5-Dodecanoylaminofluorescein di-β-D-Galactopyranoside), making it a specific substrate for β-galactosidase. The enzyme-cleaved product is retained in the cell due to the covalent binding of intracellular proteins and emits a fluorogenic signal that has an absorption/emission maxima of 490/514 nm. Cells were stained according to the manufacturer’s protocol. MIA PaCa-2 cells were seeded in a 100 mm Petri dish and exposed to UAs at IC_50_ and IC_80_ doses and gemcitabine at IC_50_ dose for up to 168 h. After incubation with examined compounds cells were trypsinized, resuspended in 1X PBS, and fixed in 4% paraformaldehyde for 15 min at room temperature, washed in 1X PBS, and then stained with the CellEvent™ Senescence Green Probe diluted 1/500 in CellEvent™ Senescence Buffer for 3 h in a 37 °C incubator with no CO_2_. Cells were washed in 1X PBS and then resuspended in 1X PBS. Data was acquired on the CytoFLEX flow cytometer (Beckman Coulter Life Sciences) and a FACS Accuri C6 flow cytometer (BD Biosciences, San Jose, CA, USA). At least 10,000 cells were collected and the results were analyzed using BD Accuri C6 Software (Version 1.0264.21) and Kaluza C software (Version 1.1. Beckmann Coulter Inc.). Mean fluorescence intensity (MFI) values were calculated as fold change compared with non-treated, stained controls. The experiment was performed at least three times.

#### Colony formation assay

The ability of MIA PaCa-2 cells to return to proliferation after UAs treatment was examined by performing a colony formation assay. MIA PaCa-2 cells were seeded in 6-well plates and exposed to UAs at IC_50_ and IC_80_ doses and gemcitabine at IC_50_ dose for up to 168 h. After compounds treatment MIA PaCa-2 cells were harvested, and 250 cells per well were seeded in a new 6-well plate with fresh medium. Cells were incubated for 14 days and then fixed with 95% ethanol, stained with Giemsa stain (Sigma-Aldrich, St Louis, MO, USA, product cat. no. GS500), photographed, and counted. The experiment was performed at least three times.

#### Western blot analysis

Panc-1, MIA PaCa-2, AsPC-1, and BxPC-3 cells were seeded and treated with test compounds analogous to cell cycle analysis. After exposure to the compounds, cells were scraped, harvested together with floating cells, and washed twice with cold 1X PBS. Cells were suspended in RIPA buffer (Abcam, Cambridge, UK, product cat. no. ab156034) with a cocktail of protease (cOmplete Tablets, Roche, Mannheim, Germany, product cat. no. 04693124001), and phosphatase inhibitors (PhosSTOP, Roche, Mannheim, Germany, product cat. no. 049906837001), and 1 mM phenylmethanesulfonyl fluoride (PMSF, Sigma-Aldrich, St Louis, MO, USA, product cat. no. 78830). The cell suspension was maintained on ice for 20 min with brief vortexing every 5 min. The lysates were then centrifuged at 14,000 g for 15 min at 4 °C. Protein concentration was determined using the DC Protein Assay (Bio-Rad, Hercules, CA, USA, product cat. no. 5000113 and no. 5000114). Samples were mixed with Laemmli buffer (Bio-Rad, Hercules, CA, USA, product cat. no. 161–0737) and 2-Mercaptoethanol (Sigma-Aldrich, St. Louis, CA, USA, product cat. no. M3148), then denatured at 100 °C for 5 min. Then 30 µg of total protein was subjected to SDS-PAGE and transfer onto nitrocellulose membranes was performed using a semi-dry blotting apparatus (Bio-Rad, Hercules, CA, USA). The membrane was blocked with every blot-blocking buffer (Bio-Rad, Hercules, CA, USA, product cat. no. 12010020), washed five times with TBST buffer, and probed with primary antibodies. Rabbit anti-c-Myc (1:2000, product cat. no. 9402), mouse anti-p21 (1:1000, product cat. no. 2946), and rabbit anti-p16 (1:1000, product cat. no. 80772) antibodies were purchased from Cell Signaling Technology (Beverly, MA, USA) and mouse anti-SMAD4 (1:250, product cat. no. sc-7966) was purchased from Santa Cruz Biotechnology (Dallas, TX, USA). Mouse anti-p53 (1:2000, product cat. no. P6874) and anti-β-actin (1:10,000, product cat. no. A5441) antibodies were purchased from Sigma-Aldrich (St. Louis, CA, USA). Secondary anti-mouse (1:2000, product cat. no. 7076) and anti-rabbit (1:2000, product cat. no. 7074) antibodies linked to horseradish peroxidase were purchased from Cell Signaling Technology (Beverly, MA, USA). To reprobe the blot with another antibody, the membrane was washed 5 times with TBST after developing the first antibody. The membrane was then treated with Restore PLUS Western Blot Stripping Buffer (Thermo Scientific, Waltham, MA, USA, product cat. no. 46430) for 15 min with gentle shaking. Next, the membrane was washed with TBST, again blocked with blocking buffer, washed, and probed with another primary antibody as described above. For each Western blot, chemiluminescence detection was performed using enhanced chemiluminescence system reagents (Thermo Scientific, Waltham, MA, USA, product cat. no. 34095). Densitometric analysis was performed using Image Lab software (Bio-Rad, Hercules, CA, USA). The experiment was performed at least three times.

#### Statistical analysis

All data are presented as median and interquartile ranges (IQR). The normality of data was assessed using the D’Agostino-Pearson test. Statistical analysis was performed using the Kruskal–Wallis one-way analysis of variance for non-parametric data and Dunn’s multiple comparison tests due to the non-normal data distribution. Differences *p* < 0.05 between the group of untreated cells (negative control) and the group of cells treated with the compound were considered statistically significant according to the following criteria: * *p* < 0.05, ** *p* < 0.01, and *** *p* < 0.001. Statistical analysis of the data was performed using GraphPad Prism 5 and 8 (GraphPad Software, San Diego, CA, USA).

## Results

### Cell viability

To examine the effects of unsymmetrical bisacridines and irinotecan or gemcitabine as positive controls on the viability of Panc-1, MIA PaCa-2, AsPC-1, and BxPC-3 cells, the MTT assay was used. Cells were treated with the studied compounds for 72 h in the concentration range 0.00001–5 µM for UAs and 0.0001–100 µM for the positive control. UAs showed a dose-dependent antiproliferative effect, and the determined IC_50_ and IC_80_ values are shown in Fig. 1. The growth inhibition curves are presented in Figs. S1–S4 in the Supplementary Information.

All three UAs strongly inhibited the growth of the four types of pancreatic cancer cells (Fig. 1b). The concentration of UAs that inhibited the growth of pancreatic cancer cells by 50% (IC_50_) did not exceed 0.198 µM, whereas the concentration that inhibited growth by 80% (IC_80_) reached a maximum of 0.486 µM. The C-2028 derivative was the most active against all tested cells; for Panc-1, MIA PaCa-2, and BxPC-3 cells, the IC_50_ (0.023–0.024 µM) and IC_80_ (0.042–0.043 µM) values were almost identical, whereas for AsPC-1 cells, the IC_50_ and IC_80_ values reached 0.027 µM and 0.102 µM, respectively. C-2045 was the least active derivative, whereas C-2053 presented IC_50_ and IC_80_ values that were more similar to those of C-2045.

Gemcitabine, a commonly used compound in the treatment of pancreatic cancer, was chosen as the positive control for MIA PaCa-2, AsPC-1, and BxPC-3 cells. Because this compound inhibited the proliferation of the abovementioned cells by a maximum of 60% relative to the control (Figs. S2–S4 in the Supplementary Information), the IC_50_ dose was chosen for further studies (Fig. 1b). For Panc-1 cells, it was not possible to determine the IC_50_ dose of gemcitabine (Fig. S1 in the Supplementary Information), as the compound inhibited cell proliferation by a maximum of 30% relative to the control. Therefore, irinotecan was chosen as the positive control for Panc-1 cells due to its lowest dose (8.5 µM), which inhibited cell growth by 50% (Fig. 1b).

The beneficial properties of UAs were confirmed by their lower toxicity toward hTERT-HPNE normal pancreatic cells (Table S1 in the Supplementary Information). The most active derivative against pancreatic cancer cells, C-2028 was less active against normal cells, and the IC_80_ had a value of 0.366 µM. Moreover, the C-2045 and C-2053 derivatives, which reached greater values than C-2028 in cancer cells, had at least two times higher IC_80_ doses in normal cells, 0.830 and 0.853 µM for C-2045 and C-2053, respectively.

### Cell cycle distribution

**Fig. 2:**
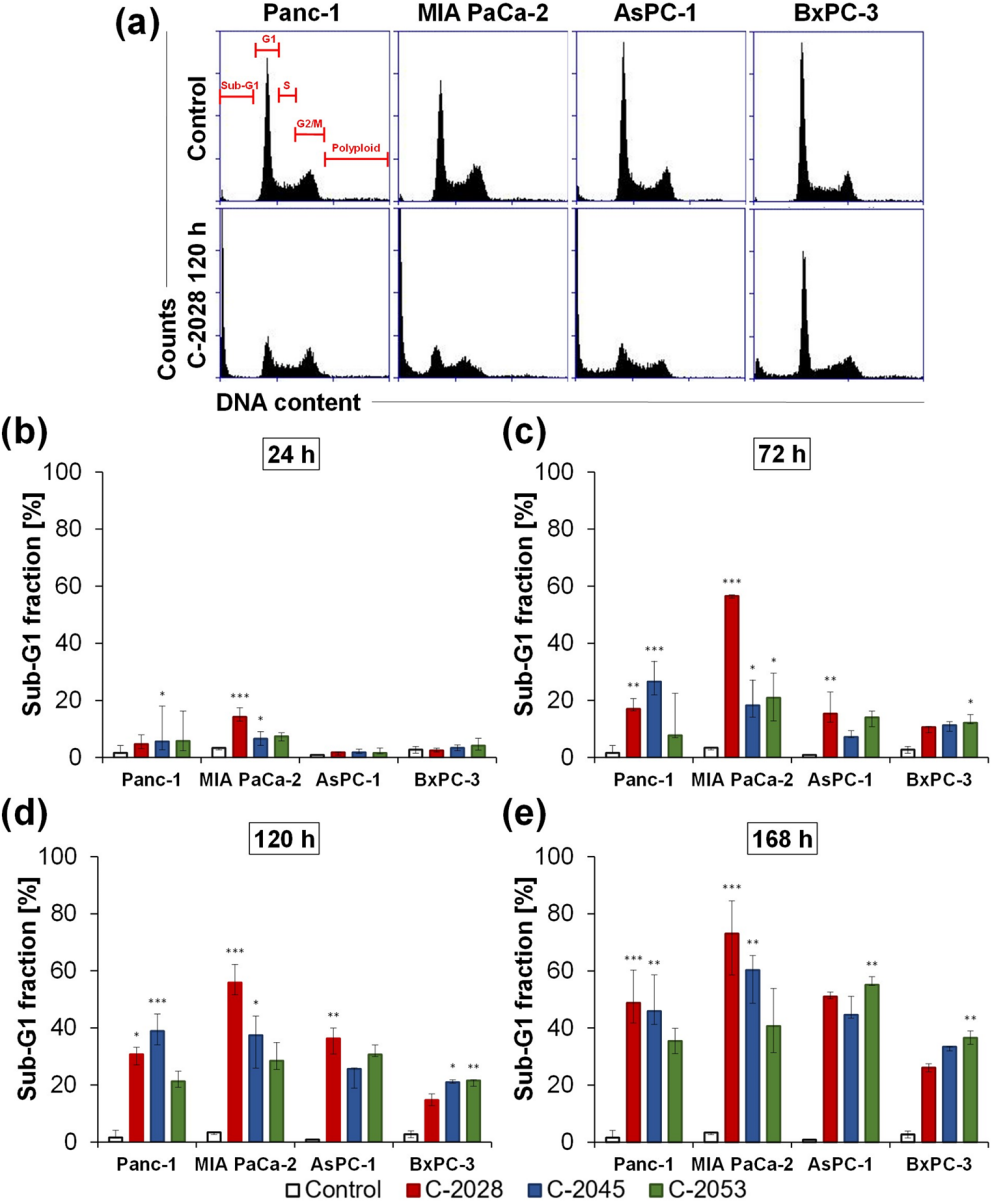
Cell cycle distribution. Panc-1, MIA PaCa-2, AsPC-1, and BxPC-3 cells were incubated with IC_80_ doses of UAs for 24, 72, 120, and 168 h. After propidium iodide (PI)/ribonuclease (RNase) staining, cells were analyzed by flow cytometry. (**a**) Representative histograms show changes in the PI-signal - DNA content (x-axis) versus cell number (y-axis) in cells after 120 h exposure to the C-2028. (**b-e**) Bar graphs represent data as the median with an interquartile range. Statistical analysis was performed using the Kruskal–Wallis test for non-parametric data and Dunn’s post-hoc test. Significantly different from the control at: * *p* < 0.05, ** *p* < 0.01, *** *p* < 0.001; *n* ≥ 4

The promising results of the antiproliferative activity of UAs against the four pancreatic cancer cell lines prompted us to further study the cellular effects induced by these compounds. First, cytometric analysis of the cell cycle distribution of pancreatic cancer cells exposed to UAs at IC_80_ doses for 24, 72, 120, and 168 h was performed, and the data are presented in Fig. 2; Table 1, and Tables S2–S5 in the Supplementary Information.

**Table 1:** The results of statistical analysis for cell cycle analysis (sub-G1 fraction) of pancreatic cancer cells after treatment with UAs at IC_80_ doses (Fig. 2), including a profile of H-values and p-levels of significance from Kruskal-Wallis test and Dunn’s multiple comparisons test. N– sample size; H– value of Kruskal-Wallis test; n/a– not applicable; *p*– significance level; * *p* < 0.05, ** *p* < 0.01, *** *p* < 0.001

			*N*	Kruskal-Wallis test		Dunn’s post hoc test (Control vs. UA)				*N*	Kruskal-Wallis test		Dunn’s post hoc test (Control vs. UA)
				H	* p-value*	*p-value*					H	*p-value*	*p-value*
24 h	Panc-1	Control	9	8.8	0.033 *		**72 h**	Panc-1	Control	9	23.5	< 0.001 ***	
		C-2028	6			0.151			C-2028	7			0.007 **
		C-2045	7			0.020 *			C-2045	7			< 0.001 ***
		C-2053	7			0.113			C-2053	7			0.052
	MIA PaCa-2	Control	8	19.1	< 0.001 ***			MIA PaCa-2	Control	8	22.1	< 0.001 ***	
		C-2028	5			< 0.001 ***			C-2028	5			< 0.001 ***
		C-2045	6			0.048 *			C-2045	7			0.021 *
		C-2053	4			0.078			C-2053	8			0.016 *
	AsPC-1	Control	3	4.7	0.209	n/a		AsPC-1	Control	3	10.6	< 0.001 ***	
		C-2028	3			n/a			C-2028	3			0.010 **
		C-2045	3			n/a			C-2045	5			> 0.999
		C-2053	3			n/a			C-2053	3			0.074
	BxPC-3	Control	4	1.9	0.636	n/a		BxPC-3	Control	4	9.6	0.004 **	
		C-2028	4			n/a			C-2028	3			0.391
		C-2045	4			n/a			C-2045	4			0.054
		C-2053	4			n/a			C-2053	3			0.0124 *
120 h	Panc-1	Control	9	21.0	< 0.001 ***		**168 h**	Panc-1	Control	9	19.5	< 0.001 ***	
		C-2028	4			0.012 *			C-2028	6			< 0.001 ***
		C-2045	4			< 0.001 ***			C-2045	5			0.002 **
		C-2053	4			0.244			C-2053	4			0.378
	MIA PaCa-2	Control	8	26.3	< 0.001 ***			MIA PaCa-2	Control	8	21.7	< 0.001 ***	
		C-2028	6			< 0.001 ***			C-2028	7			< 0.001 ***
		C-2045	6			0.015 *			C-2045	6			0.002 **
		C-2053	7			0.068			C-2053	7			0.127
	AsPC-1	Control	3	14.0	0.007 **			AsPC-1	Control	3	9.7	< 0.001 ***	
		C-2028	5			0.006 **			C-2028	3			0.210
		C-2045	3			> 0.999			-2045	3			0.639
		C-2053	3			0.121			C-2053	3			0.007 **
	BxPC-3	Control	4	15.9	0.003 **			BxPC-3	Control	4	13.2	< 0.001 ***	
		C-2028	4			0.618			C-2028	4			0.618
		C-2045	3			0.022 *			C-2045	3			0.084
		C-2053	4			0.006 **			C-2053	4			0.002 **

The most important change in the cell cycle of the studied pancreatic cancer cells induced by UAs was a time-dependent increase in the population of hypodiploid cells (DNA content < 2 N, sub-G1 fraction), indicating that the cells were undergoing cell death. Other alternations in cell cycle progression, such as the accumulation of cells in a particular phase of the cycle, were not observed. Only up to 120 h of incubation, a slight increase in the polyploid cell population was observed.

In Panc-1 cells, after 168 h of incubation, the percentages of hypodiploid cells reached 50.7, 51.1, and 35.5% for C-2028, C-2045, and C-2053, respectively. In MIA PaCa-2 cells, the sub-G1 population reached the highest level following 168 h of C-2028 treatment (70.7%). In turn, for C-2045 and C-2053, the percentages of cells with DNA < 2 N reached 58.4 and 49.0%, respectively. In AsPC-1 cells, the percentage of the sub-G1 fraction at 168 h reached maximum levels of 51.3, 46.4, and 56.0% for C-2028, C-2045, and C-2053, respectively. In turn, treatment of BxPC-3 cells with UAs led to an increase in the percentage of cells with fragmented DNA; however, after 168 h, the sub-G1 fraction reached the lowest percentages among all the lines tested, with 26.1, 32.9, and 36.6% for C-2028, C-2045, and C-2053, respectively.

**Fig. 3:**
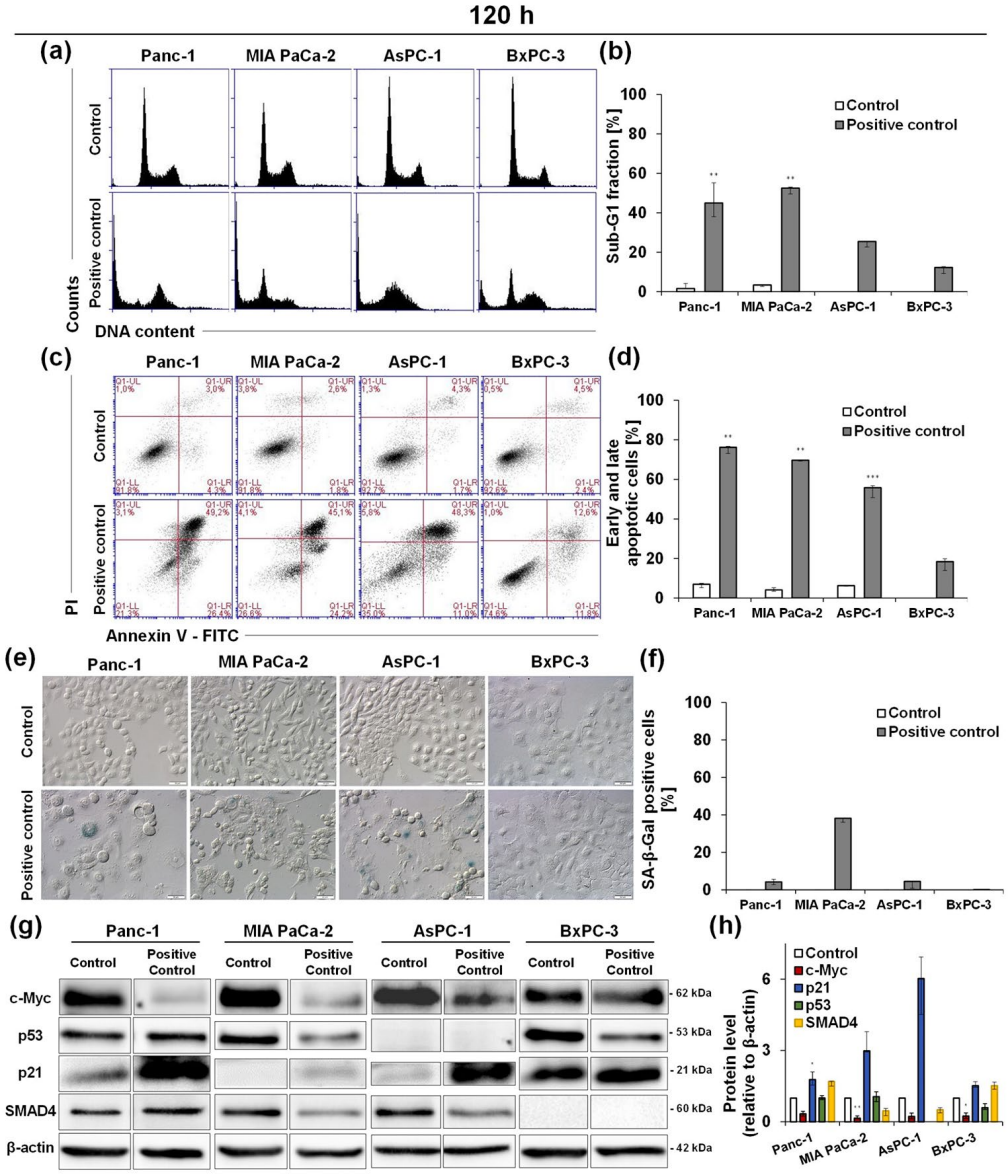
Effect of the positive control drugs on pancreatic cancer cells. Application of irinotecan (Panc-1 cells) and gemcitabine (MIA PaCa-2, AsPC-1 and BxPC-3 cells) at IC_50_ doses to study the following characteristics: (**a**,**b**) cell cycle distribution, (**c**,**d**) cell membrane integrity, (**e**,**f**) induction of accelerated senescence, and (**g**,**h**) levels of c-Myc, p53, p21 and SMAD4 proteins. (**a**) Representative histograms show changes in the propidium iodide (PI)-signal– DNA content (x-axis) versus cell number (y-axis) analyzed by flow cytometry. (**c**) Representative cytograms show the annexin V- FITC (**a**) signal versus the PI signal analyzed by flow cytometry. Viable cells– A-PI-; early apoptotic cells– A+/PI-; late apoptotic cells– A+/PI+; necrotic cells– A-/PI+. (**e**) Representative images of microscopic observation of accelerated senescence. Cells were fixed, incubated with X-gal (substrate for β-galactosidase) staining solution, and observed under light microscopy using the Nomarski interference contrast at 200× magnification, scale bar 50 μm. Blue stain indicates senescence-associated (SA)-β-galactosidase activity. (**g**) Western blot analysis of c-Myc, p53, p21, and SMAD4 in pancreatic cancer cells. Whole-cell extracts (30 µg of protein/lane) were prepared, separated by polyacrylamide gel electrophoresis, and transferred to the membrane by the semi-dry method. Immunoblotting and enhanced chemiluminescence (ECL) development were performed to determine protein levels by densitometric analysis. (**b**,**d**,**f**,**h**) The bar graphs show the median and interquartile range of indicated parameters. FITC– fluorescein isothiocyanate; SA-β-Gal– senescence-associated-β-galactosidase. Statistical analysis was performed using the Kruskal–Wallis test for non-parametric data and Dunn’s post-hoc test. Significantly different from the control at: * *p* < 0.05, ** *p* < 0.01, *** *p* < 0.001; *n* ≥ 3

Irinotecan (positive control) caused an increase in the number of Panc-1 cells in the sub-G1 phase, which reached 46.0% after 120 h of incubation. (Fig. 3a–b; Table 2, and Table S2 in the Supplementary Information). Another positive control, gemcitabine, also induced time-dependent increases in the hypodiploid DNA content in pancreatic cells (MIA PaCa-2, AsPC-1, and BxPC-3 cells); however, this increase was less pronounced in AsPC-1 and BxPC-3 cells than following UAs treatment (Fig. 3a–b; Table 2, and Tables S3–S5 in the Supplementary Information).

**Table 2:** The results of statistical analysis for cell cycle analysis (sub-G1 fraction) of pancreatic cancer cells after treatment with positive controls (+): irinotecan (IR) or gemcitabine (GEM) (Fig. 3b), including a profile of H-values and p-levels of significance from Kruskal-Wallis test and Dunn’s multiple comparisons test. N– sample size; H– value of Kruskal-Wallis test; *p*– significance level; * *p* < 0.05, ** *p* < 0.01, *** *p* < 0.001

			Kruskal-Wallis test		Dunn’s post hoc test (Control vs. (+))
		N	H	*p-value*	*p-value*
Panc-1	Control	9	21.0	< 0.001 ***	
	IR	3			0.001 **
MIA PaCa-2	Control	8	26.3	< 0.001 ***	
	GEM	4			0.001 **
AsPC-1	Control	3	14.0	0.007 **	
	GEM	3			> 0.99
BxPC-3	Control	4	15.9	0.003 **	
	GEM	4			0.840

### Induction of apoptosis

**Fig. 4:**
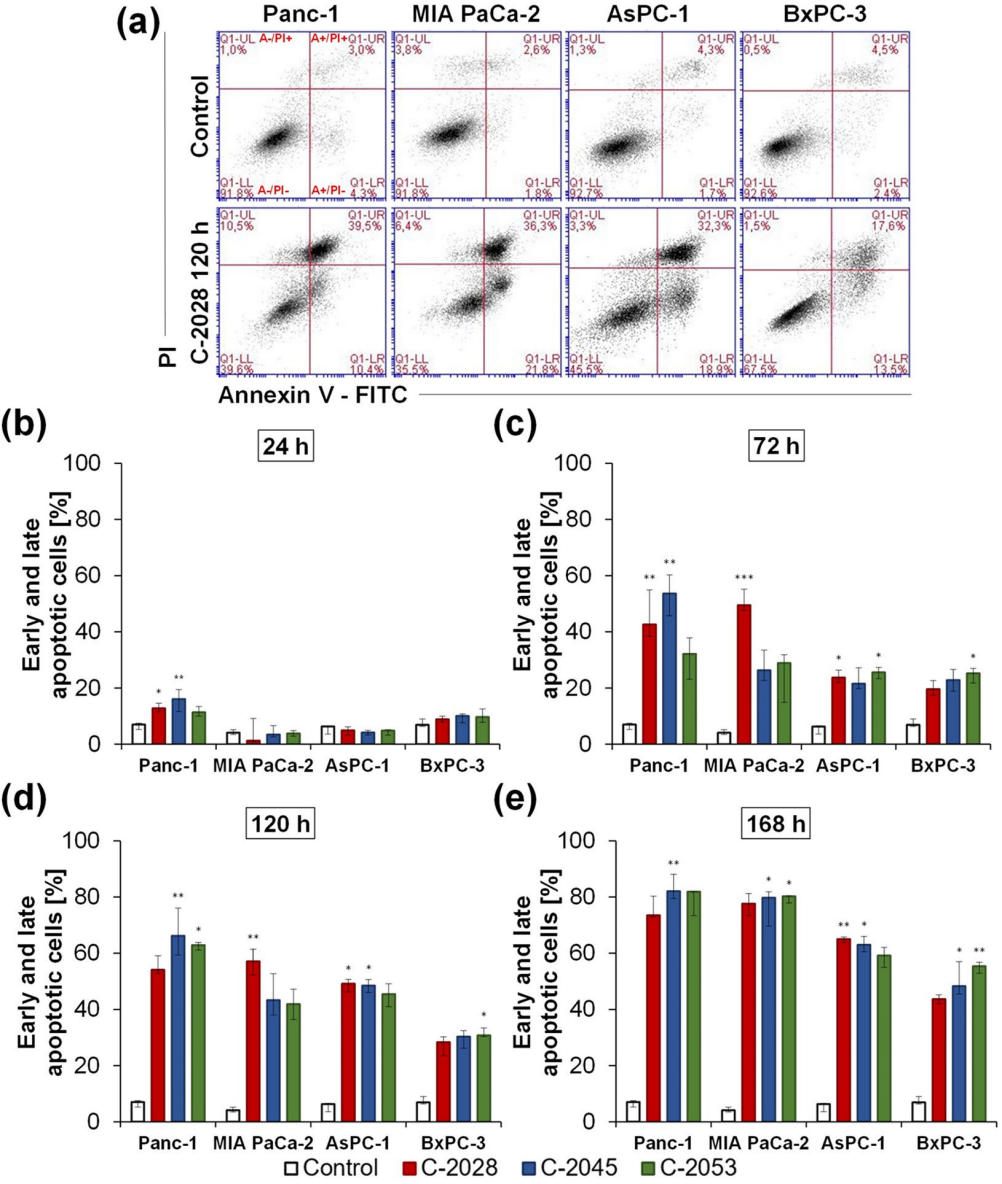
Phosphatidylserine translocation and membrane integrity. Panc-1, MIA PaCa-2, AsPC-1, and BxPC-3 cells were incubated with IC_80_ doses of UAs for 24, 72, 120, and 168 h. After annexin V-FITC (**a**) and propidium iodide (PI) staining, cells were analyzed by flow cytometry. (**a**) Representative cytograms show the annexin V- FITC signal versus the PI signal in cells after 120 h exposure to the C-2028. Viable cells– A-PI-; early apoptotic cells– A+/PI-; late apoptotic cells– A+/PI+; necrotic cells– A-/PI+. (**b-e**) Bar graphs represent data as the median with an interquartile range. FITC– fluorescein isothiocyanate. Statistical analysis was performed using the Kruskal–Wallis test for non-parametric data and Dunn’s post-hoc test. Significantly different from the control at: * p < 0.05, ** p < 0.01, *** p < 0.001; *n* ≥ 4

To determine the type of cell death caused by the tested compounds, an analysis of cell membrane asymmetry and integrity was performed using annexin V-FITC and PI dual staining. Panc-1, MIA PaCa-2, AsPC-1, and BxPC-3 cells were treated with UAs at IC_80_ doses for 24, 72, 120, and 168 h. The cell membrane statuses of pancreatic cancer cells exposed to UAs are presented in Fig. 4; Table 3, and Tables S6–S9 in the Supplementary Information.

**Table 3:** The results of statistical analysis for phosphatidylserine translocation and membrane integrity analysis (early- and late apoptotic cells) of pancreatic cancer cells after treatment with UAs at IC_80_ doses (Fig. 4), including a profile of H-values and p-levels of significance from Kruskal-Wallis test and Dunn’s multiple comparisons test. N– sample size; H– value of Kruskal-Wallis test; n/a– not applicable; *p*– significance level; * *p* < 0.05, ** *p* < 0.01, *** *p* < 0.001

			*N*	Kruskal-Wallis test		Dunn’s post hoc test (Control vs. UA)				*N*	Kruskal-Wallis test		Dunn’s post hoc test (Control vs. UA)
				H	*p-value*	*p-value*					H	*p-value*	*p-value*
24 h	Panc-1	Control	5	11.6	0.001 **		**72 h**	Panc-1	Control	5	15.0	0.0018 **	
		C-2028	4			0.036 *			C-2028	6			0.007 **
		C-2045	4			0.005 **			C-2045	4			0.002 **
		C-2053	4			0.195			C-2053	4			0.625
	MIA PaCa-2	Control	5	0.8	0.847			MIA PaCa-2	Control	5	14.8	< 0.001 ***	
		C-2028	5			n/a			C-2028	5			< 0.001 ***
		C-2045	5			n/a			C-2045	4			0.208
		C-2053	5			n/a			C-2053	4			0.208
	AsPC-1	Control	4	2.1	0.581			AsPC-1	Control	4	9.7	0.0064 **	
		C-2028	4			n/a			C-2028	4			0.043 *
		C-2045	4			n/a			C-2045	4			0.224
		C-2053	4			n/a			C-2053	4			0.011 *
	BxPC-3	Control	3	4.4	0.230			BxPC-3	Control	3	9.3	0.008 **	
		C-2028	4			n/a			C-2028	4			0.459
		C-2045	4			n/a			C-2045	4			0.069
		C-2053	4			n/a			C-2053	4			0.011 *
120 h	Panc-1	Control	5	14.5	0.006 **		**168 h**	Panc-1	Control	5	10.4	0.001 **	
		C-2028	3			0.571			C-2028	3			0.305
		C-2045	3			0.010 **			C-2045	3			0.010 **
		C-2053	3			0.027 *			C-2053	3			0.066
	MIA PaCa-2	Control	5	15.6	0.004 **			MIA PaCa-2	Control	5	10.6	0.004 **	
		C-2028	4			0.002 **			C-2028	4			0.091
		-2045	3			0.157			C-2045	4			0.024 *
		C-2053	3			0.377			C-2053	5			0.013 *
	AsPC-1	Control	4	14.6	0.006 **			AsPC-1	Control	4	11.4	< 0.001 ***	
		C-2028	4			0.02 *			C-2028	4			0.004 **
		C-2045	4			0.025 *			C-2045	4			0.043 *
		C-2053	4			0.2235			C-2053	4			0.413
	BxPC-3	Control	3	14.6	0.006 **			BxPC-3	Control	4	11.5	< 0.001 ***	
		C-2028	4			0.493			-2028	4			0.618
		C-2045	4			0.058			C-2045	3			0.084 *
		C-2053	4			0.013 *			C-2053	4			0.002 **

In Panc-1 cells, all three UA derivatives caused significant changes (Table 3) in the asymmetry and integrity of the cell membrane, which indicated the induction of cell death. After 168 h of incubation, the number of viable cells (A-/PI-) decreased to less than 22% for all the tested compounds. After 72 h of incubation with the C-2028 and C-2045 derivatives, annexin V-positive cells (i.e., early and late apoptotic cells) accounted for 42.5 and 53.2% of the total cell population, respectively. In contrast, for C-2053, this population reached 31.1%. After the longest incubation time, the percentages of apoptotic cells were similar for all the compounds and ranged from 75.6 to 87.2%. The proportions of necrotic cells for the C-2028 and C-2045 derivatives did not exceed 5.3%, whereas for C-2053, it reached the highest level of 10.2% after 72 h. In MIA PaCa-2 cells, annexin V-positive cells were found at the highest percentage after 72 h of exposure to C-2028 (51%). The apoptotic cell populations reached maximum levels after 168 h: 77.5, 77.7, and 79.4% for C-2028, C-2045, and C-2053, respectively, and greater proportions of these fractions were late-apoptotic cells. Necrotic cells accounted for the highest percentages of the tested cell populations after 72 h, reaching 11.6, 13.6, and 9.4% for C-2028, C-2045, and C-2053, respectively. The fraction of apoptotic AsPC-1 cells gradually increased, reaching 64.9, 63.1, and 58.8% after 168 h of treatment with C-2028, C-2045, and C-2053, respectively. Late apoptotic cells constituted the predominant fraction of annexin-positive cells. The population of necrotic cells did not exceed 5.5% for all the UA derivatives tested. In BxPC-3 cells, the percentages of the annexin V-positive fraction peaked after 168 h at 43.6, 50.3, and 55.0% for C-2028, C-2045, and C-2053, respectively. The proportions of early and late apoptotic cells were very similar. Conversely, necrotic cells represented very small percentages of the cell population studied, not exceeding 3.8%.

In Panc-1 cells treated with irinotecan, the positive control drug, a time-dependent increase in the number of cells stained with annexin V was observed. After 120 h of incubation, 75.3% of the cells exhibited changes in the asymmetry of the cell membrane (Fig. 3c–d; Table 4, and Table S6 in the Supplementary Information). Necrotic cells accounted for the highest percentage of the tested cell population after 72 h, reaching 13.8%. A strong effect was also triggered by gemcitabine in MIA PaCa-2 and AsPC-1 cells, where after the longest exposure time early and late apoptotic cells were 71.5 and 61.8% for each line, respectively. Compared with UAs, in BxPC-3 cells treated with gemcitabine, apoptosis was induced in the lowest population. Only 31.3% of the cells were annexin V-positive (Fig. 3c–d; Table 4 and Tables S7–S9 in the Supplementary Information). Gemcitabine caused necrosis at levels lower than 8.4% in MIA PaCa-2, AsPC-1, and BxPC-3 cells.

**Table 4:** The results of statistical analysis for phosphatidylserine translocation and membrane integrity analysis (early- and late apoptotic cells) of pancreatic cancer cells after treatment with positive controls (+): irinotecan (IR) or gemcitabine (GEM) (Fig. 3d), including a profile of H-values and p-levels of significance from Kruskal-Wallis test and Dunn’s multiple comparisons test. N– sample size; H– value of Kruskal-Wallis test; *p*– significance level; * *p* < 0.05, ** *p* < 0.01, *** *p* < 0.001

			Kruskal-Wallis test		Dunn’s post hoc test (Control vs. (+))
		N	H	*p-value*	*p-value*
Panc-1	Control	5	14.5	0.006 **	
	IR	3			0.002 **
MIA PaCa-2	Control	5	15.6	0.004 **	
	GEM	3			0.001 **
AsPC-1	Control	4	14.6	0.006 **	
	GEM	4			< 0.001 ***
BxPC-3	Control	3	14.6	0.006 **	
	GEM	4			> 0.999

### Morphological changes in the cell nuclei

Cell membrane asymmetry and integrity analysis showed a time-dependent induction of apoptosis in cells treated with UAs. To confirm the induction of this type of cell death, an analysis of cell nuclear morphology was performed using Hoechst 33,342 staining. Panc-1, MIA PaCa-2, AsPC-1, and BxPC-3 cells were treated with UAs at IC_80_ doses for 24, 72, 120, and 168 h. Cell nuclear morphology following exposure to UAs is presented in Figs. S5–S8 in the Supplementary Information.

The most profound change in the nuclear morphology of Panc-1 cells was the appearance of condensed chromatin with material collected at the edges of the nuclear envelope, which formed a small pycnotic nucleus, as well as a small group of apoptotic bodies. During incubation, the nuclei became larger, with the exception of the C-2045 derivative, and the proportion of nuclei with altered morphology increased over time. Compared with the other compounds, exposure of Panc-1 cells to the C-2045 derivative resulted in a greater induction of apoptosis. Mitotic catastrophe, which is characterized by the presence of multinucleated cells, was also confirmed, but in a small population of cells. The nuclei of MIA PaCa-2 cells treated with UAs also underwent numerous changes. All the tested compounds induced apoptosis in a large population of cells, as indicated by the multiple cell nuclei that exhibited apoptotic features, the most profoundly after C-2028 exposure. In addition, compared with control cells, exposure of MIA PaCa-2 cells to UAs led to the enlargement of cell nuclei. This effect was mainly observed after 120 h of incubation. Mitotic catastrophe and abnormal mitosis were noted in some of the cell nuclei. In AsPC-1 cells, the strongest induction of apoptosis was observed after exposure to C-2028 and C-2053, with the greatest increase occurring after 120 h of incubation. However, apoptotic bodies were present after 24 h of UA exposure. At 168 h of incubation, some nuclei were significantly enlarged, and a few polyploid cells were also observed, as were cells in which mitosis was abnormal. The main change in the morphology of BxPC-3 cell nuclei was the presence of nuclei with condensed chromatin and apoptotic bodies. The cell size did not change significantly with incubation time, except in cells exposed to the C-2045 derivative, where the number of nuclei was slightly greater after 72 and 120 h of incubation. Other observed abnormalities included multinucleated cells and abnormal mitosis, but these affected only single cells. The greatest changes indicating apoptosis were triggered in BxPC-3 cells by the C-2053 derivative after 168 h of exposure.

The positive control drugs also triggered many changes in PC cell nuclear morphology (Figs. S5–S8 in the Supplementary Information). After treatment with irinotecan, Panc-1 cells exhibited features characteristic of apoptosis and, to a lesser extent, aberrant mitosis and mitotic catastrophe. Numerous cell nuclei became enlarged. Gemcitabine induced apoptosis in MIA PaCa-2 cells and AsPC-1 cells, and some of the cells underwent aberrations during mitosis. However, cells with normal morphology of nuclei were still observed. Moreover, the nuclei of BxPC-3 cells did not change much after exposure to gemcitabine. Only a few cells exhibited features of abnormal mitosis or mitotic catastrophe.

### Detection of accelerated senescence

Previous data have shown that a fraction of pancreatic cancer cells remain viable even after long incubation times with IC_80_ doses of UAs. Therefore, the process of accelerated senescence was investigated following UAs treatment depending on the dose used. In senescent cells, the activity of the SA-β-galactosidase enzyme released into the cytoplasm is increased. Its level can be examined by treating cells with an X-gal staining solution, which, at pH 6.0, is metabolized by cytosolic SA-β-galactosidase to a blue insoluble compound, as observed under a light microscope.

**Fig. 5:**
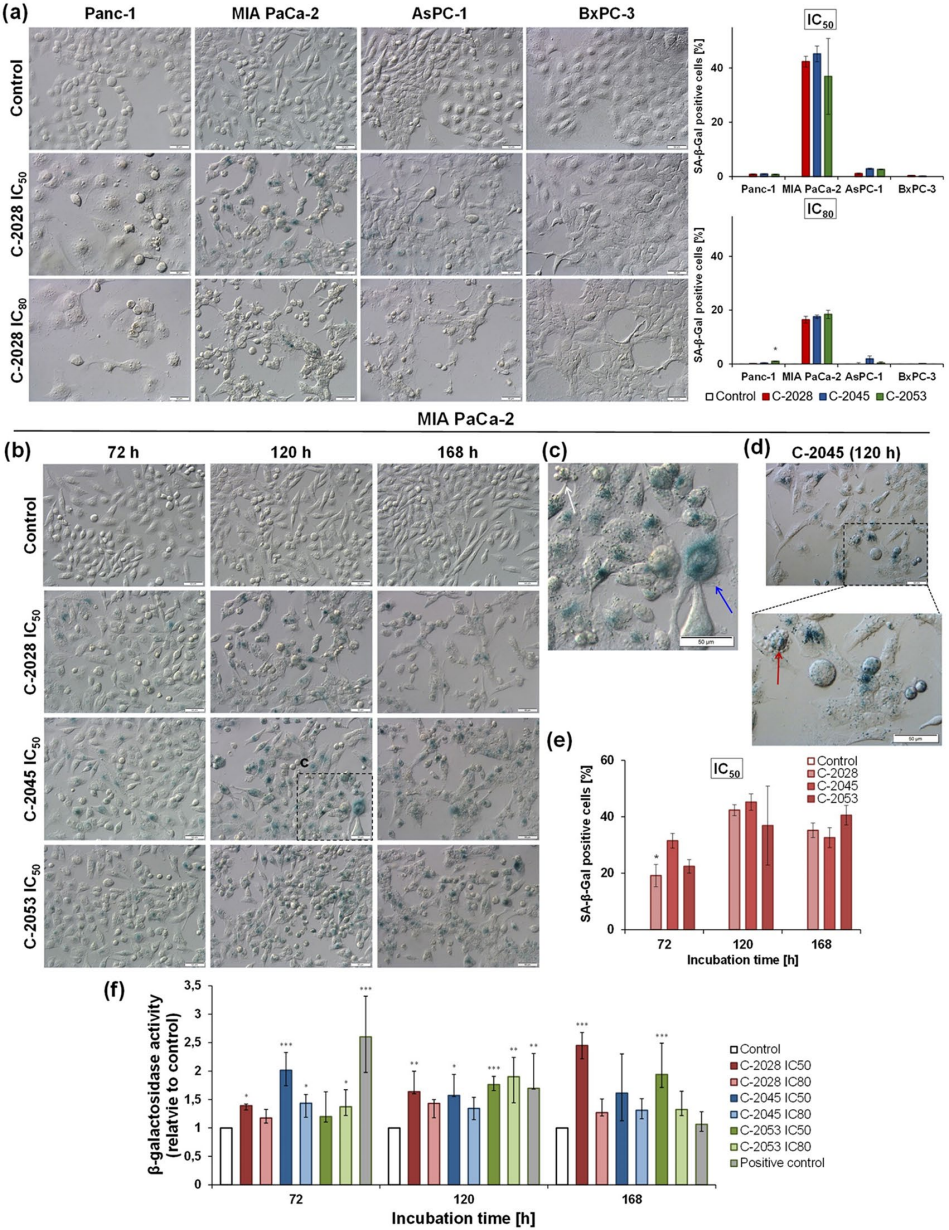
Accelerated senescence detection. (**a**) Induction of accelerated senescence in Panc-1, MIA PaCa-2, AsPC-1, and BxPC-3 cells by C-2028 at IC50 and IC80 doses after 120 h of incubation. Graphs show the median with an interquartile range of SA-β-Gal (senescence-associated-β-galactosidase) positive cells percentage from the microscopic analysis. (**b-d**) Representative images of MIA PaCa-2 cells after treatment with IC50 doses of UAs for 72, 120, and 168 h. (**c**) Enlarged fragment of the selected image, the blue arrow indicates a senescent cell and the white arrow indicates an apoptotic cell. (**d**) The red arrow indicates an apoptotic cell with a blue stain. (**e**) Graphs represent the median with an interquartile range of SA-β-Gal positive cells percentage from the microscopic analysis. Briefly, cells were fixed, incubated with X-gal (substrate for β-galactosidase) staining solution, and observed under light microscopy using the Nomarski interference contrast at 200× magnification, scale bar 50 μm. (**f**) Changes of the SA-β-Gal activity in MIA PaCa-2 cells after 72, 120, and 168 h of incubation with IC50 and IC80 doses of UAs and positive control (gemcitabine) in comparison to untreated control cells SA-β-Gal activity was estimated by flow cytometry method as mean fluorescence intensity (MFI). Statistical analysis was performed using the Kruskal–Wallis test for non-parametric data and Dunn’s post-hoc test. Significantly different from the control at: * p < 0.05, ** p < 0.01, *** p < 0.001; *n* ≥ 2

Microscopic observation of Panc-1, MIA PaCa-2, AsPC-1, and BxPC-3 cells treated with UAs at IC_80_ and IC_50_ doses for 72, 120, and 168 h revealed that the IC_50_ doses of the compounds triggered accelerated senescence to a greater extent in MIA PaCa-2 cells (Fig. 5a; Table 5). Blue staining was observed after 72, 120, and 168 h of MIA PaCa-2 cell exposure to UAs at the IC_50_ dose (Fig. 5b–e; Table 6). The greatest number of senescent cells was observed after 120 h of incubation with the C-2028 and C-2045 derivatives (Fig. 5e; Table 6). Moreover, these cells were enlarged, flat, and granular. After 168 h of incubation, the cell population was reduced; however, some of the cells exhibited apoptotic cell morphology (shrunken cells with characteristic blebs) and were stained blue (a product of SA-β-galactosidase activity). These findings suggest that senescent cells may undergo apoptosis, as shown in Fig. 5d, where the red arrow indicates an apoptotic cell with blue staining. Detailed pictures of accelerated senescence observations in all tested pancreatic cancer cells treated with UAs can be found in Figs. S9–S12 in the Supplementary Information. In Panc-1 cells treated only with C-2053 at the IC_80_ dose, single cells with characteristic blue staining were observed after 120 and 168 h of exposure. In AsPC-1 cells, the blue indicator was present only in single cells treated with UAs at the IC_50_ dose and with gemcitabine (Fig. 3e–f; Table 7). Microscopic observation of BxPC-3 cells revealed no induction of accelerated senescence by UAs (Fig. 5a and Fig. S12 in the Supplementary Information). Only single cells with blue staining were observed after exposure to gemcitabine (Fig. 3e–f; Table 7). Despite the presence of single cells with characteristic blue staining in Panc-1, AsPC-1, and BxPC-3 cells, only the MIA PaCa-2 cells underwent accelerated cellular senescence. Moreover, this process occurred in a dose-dependent manner, especially in cells treated with UAs at the IC_50_ dose.

**Table 5:** The results of statistical analysis for microscopic detection of accelerated senescence (% of SA-β-gal positive cells) in pancreatic cancer cells after treatment with C-2028 at IC_50_ and IC_80_ doses (Fig. 5a), including a profile of H-values and p-levels of significance from Kruskal-Wallis test and Dunn’s multiple comparisons test. N– sample size; H– value of Kruskal-Wallis test; n/a– not applicable; *p*– significance level; * *p* < 0.05, ** *p* < 0.01, *** *p* < 0.001

			*N*	Kruskal-Wallis test		Dunn’s post hoc test (Control vs. UA)				*N*	Kruskal-Wallis test		Dunn’s post hoc test (Control vs. UA)
				H	*p-valu*e	*p-value*					H	*p-value*	*p-value*
IC_50_	Panc-1	Control	2	7.4	0.052		**IC**_**80**_	Panc-1	Control	2	7.5	0.012 *	
		C-2028	2			n/a			C-2028	2			0.658
		C-2045	2			n/a			C-2045	2			0.256
		C-2053	2			n/a			C-2053	2			0.021 *
	MIA PaCa-2	Control	2	5.8	0.216			MIA PaCa-2	Control	2	4.7	0.238	
		C-2028	2			n/a			C-2028	2			n/a
		C-2045	2			n/a			C-2045	2			n/a
		C-2053	2			n/a			C-2053	2			n/a
	AsPC-1	Control	2	7.7	0.048 *			AsPC-1	Control	2	3.1	0.429	
		C-2028	2			> 0.999			C-2028	2			n/a
		C-2045	2			0.072			C-2045	2			n/a
		C-2053	2			0.194			C-2053	2			n/a
	BxPC-3	Control	2	4.9	0.429			BxPC-3	Control	2	3.0	> 0.999	
		C-2028	2			n/a			C-2028	2			n/a
		C-2045	2			n/a			C-2045	2			n/a
		C-2053	2			n/a			C-2053	2			n/a

**Table 6:** The results of statistical analysis for microscopic detection of accelerated senescence (% of SA-β-gal positive cells) in MIA PaCa-2 cells after treatment with UAs at IC_50_ doses (Fig. 5e), including a profile of H-values and p-levels of significance from Kruskal-Wallis test and Dunn’s multiple comparisons test. N– sample size; H– value of Kruskal-Wallis test; n/a– not applicable; *p* - significance level; * *p* < 0.05, ** *p* < 0.01, *** *p* < 0.001

			*N*	Kruskal-Wallis test		Dunn’s post hoc test (Control vs. UA)
				H	*p-value*	*p-value*
MIA PaCa-2 IC_50_	72 h	Control	2	6.2	0.029 *	
		C-2028	2			0.914
		C-2045	2			0.041 *
		C-2053	2			0.452
	120 h	Control	2	4.2	0.305	
		C-2028	2			n/a
		C-2045	2			n/a
		C-2053	2			n/a
	168 h	Control	2	5.6	0.0952	
		C-2028	2			n/a
		C-2045	2			n/a
		C-2053	2			n/a

**Table 7:** The results of statistical analysis for microscopic detection of accelerated senescence (% of SA-β-gal positive cells) in pancreatic cancer cells after treatment with positive controls (+): irinotecan (IR) or gemcitabine (GEM) (Fig. 3f), including a profile of H-values and p-levels of significance from Kruskal-Wallis test and Dunn’s multiple comparisons test. N– sample size; H– value of Kruskal-Wallis test; n/a– not applicable; *p* - significance level; * *p* < 0.05, ** *p* < 0.01, *** *p* < 0.001

			Kruskal-Wallis test		Dunn’s post hoc test (Control vs. (+))
		N	H	*p-value*	*p-value*
Panc-1	Control	2	7.4	0.052	
	IR	2			n/a
MIA PaCa-2	Control	2	5.8	0.216	
	GEM	2			n/a
AsPC-1	Control	2	7.7	0.048 *	
	GEM	3			0.071
BxPC-3	Control	2	4.9	0.429	
	GEM	2			n/a

To confirm the increased SA-β-galactosidase activity in MIA PaCa-2 cells, cytometric analysis of SA-β-galactosidase levels after exposure to UAs at the IC_80_ and IC_50_ doses for 72, 120, and 168 h was performed. SA-β-galactosidase activity is shown as a fold change in the mean fluorescence intensity (MFI) after incubation with UAs or the positive control relative to that of untreated cells (Fig. 5f; Table 8, and Table S10 in the Supplementary Information). Exposure of MIA PaCa-2 cells to UAs at the IC_50_ doses resulted in a greater increase in SA-β-galactosidase activity than treatment with the IC_80_ dose. Treatment with the C-2028 and C-2053 derivatives at the IC_50_ dose induced a gradual increase in the population of senescent cells, reaching a maximum after 168 h, with MFIs of 2.5 and 2.0, respectively. In contrast, this parameter value for the C-2045 derivative remained consistently high throughout the analysis (1.7–2.0). When MIA PaCa-2 cells were treated with compounds at the IC_80_ doses, elevated levels of SA-β-galactosidase were observed relative to those in the control, and the MFIs ranged from 1.2 to 1.9, reaching the highest values after 120 h of incubation. These results correlate with the microscopic observations, which indicated that the senescence process was most strongly induced by UAs at IC_50_ concentrations in MIA PaCa-2 cells. Upon exposure to the positive control, gemcitabine caused an increase in SA-β-galactosidase activity to the highest level after 72 h, which was more than 2.6 times greater than that in untreated cells. However, the MFI value gradually decreased with time, reaching the same level as that of the control after 168 h.

**Table 8:** The results of statistical analysis for cytometric detection of accelerated senescence (SA-β-gal activity) in MIA PaCa-2 cells after treatment with UAs at IC_50_ and IC_80_ doses (Fig. 5f), including a profile of H-values and p-levels of significance from Kruskal-Wallis test and Dunn’s multiple comparisons test. N– sample size; H– value of Kruskal-Wallis test; *p*– significance level; * *p* < 0.05, ** *p* < 0.01, *** *p* < 0.001

			*N*	Kruskal-Wallis test		Dunn’s post hoc test (Control vs. UA)
				H	*p-value*	*p-value*
MIA PaCa-2	72 h	Control	7	31.0	< 0.001 ***	
		C-2028 IC_50_	6			0.030 *
		C-2028 IC_80_	5			0.915
		C-2045 IC_50_	5			< 0.001 ***
		C-2045 IC_80_	6			0.024 *
		C-2053 IC_50_	7			0.074
		C-2053 IC_80_	6			0.024 *
		Positive control	3			< 0.001 ***
	120 h	Control	7	28.7	< 0.001 ***	
		C-2028 IC_50_	5			0.005 **
		C-2028 IC_80_	6			> 0.999
		C-2045 IC_50_	4			0.046 *
		C-2045 IC_80_	5			> 0.999
		C-2053 IC_50_	6			< 0.001 ***
		C-2053 IC_80_	5			0.004 **
		Positive control	4			0.002 **
	168 h	Control	7	32.4	< 0.001 ***	
		C-2028 IC_50_	6			< 0.001 ***
		C-2028 IC_80_	5			0.647
		C-2045 IC_50_	5			0.153
		C-2045 IC_80_	5			0.614
		C-2053 IC_50_	7			< 0.001 ***
		C-2053 IC_80_	5			0.415
		Positive control	6			> 0.999

### Ability of MIA PaCa-2 cells to return to proliferation

Because only MIA PaCa-2 cells underwent accelerated senescence, the reversibility or irreversibility of proliferation arrest was tested. The ability to permanently inhibit cell growth after drug exposure is important from an anticancer therapy perspective due to the possible disease recurrence as well as the induction of the metastatic process. MIA PaCa-2 cells were treated with UAs at the IC_80_ and IC_50_ doses for 24, 72, 120, and 168 h, and after two weeks of post-incubation, their ability to form colonies was investigated.

**Fig. 6:**
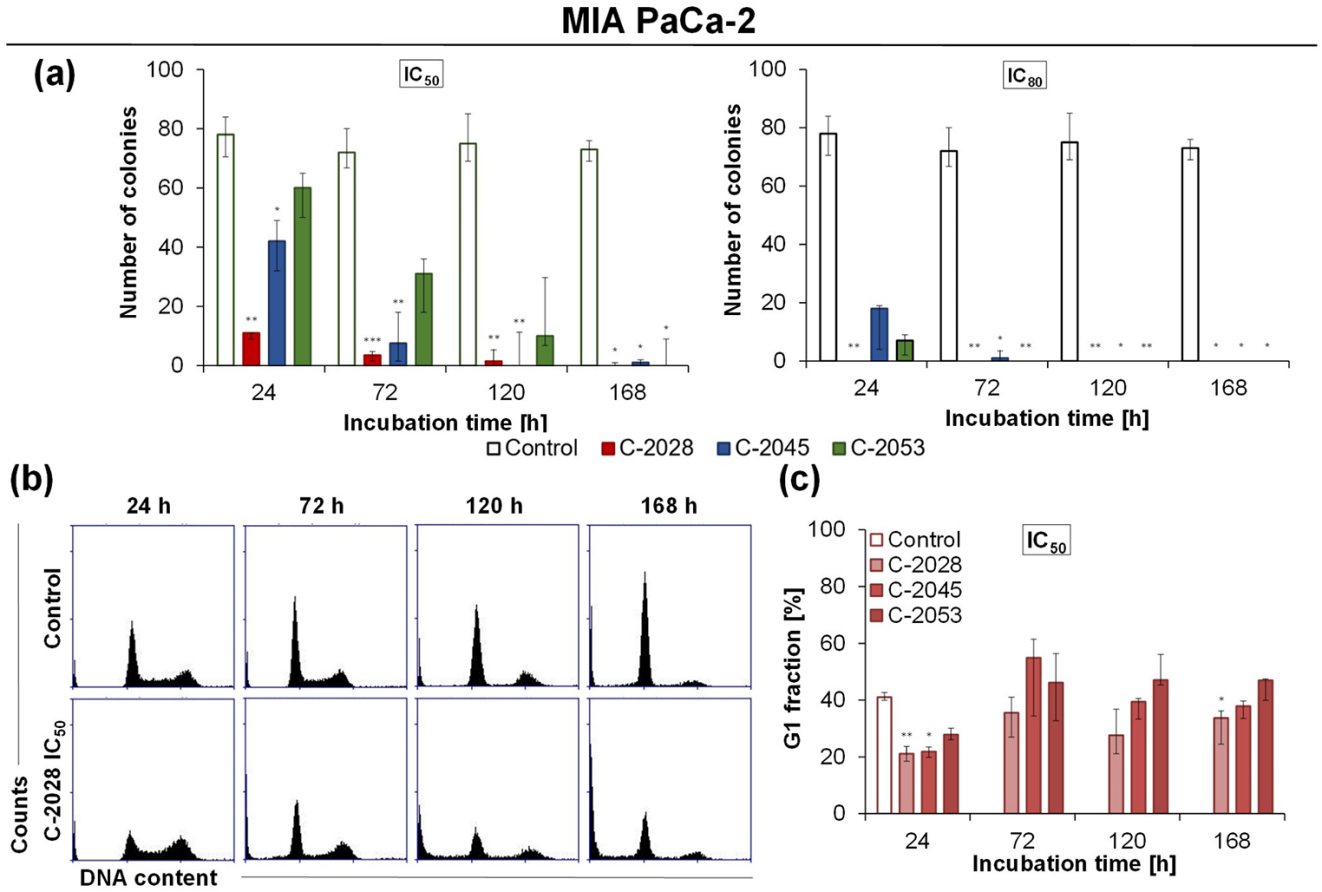
Ability of MIA PaCa-2 cells to return to proliferation after UAs exposure. (**a**) Bar graphs represent the number of colonies formed as the median with an interquartile range. Cells were exposed to IC_50_ and IC_80_ doses of UAs for 24, 72, 120, and 168 h. Next, 250 cells were seeded onto a fresh plate, post-incubated for 14 days, fixed with ethanol, stained with Giemsa dye, and counted. (**b-c**) Cell cycle distribution of MIA PaCa-2 cells after treatment with C-2028, C-2045, and C-2053. Cells were incubated with IC_50_ doses of UAs for 24, 72, 120, and 168 h. After propidium iodide (PI)/ ribonuclease (RNase) staining, cells were analyzed by flow cytometry. (**b**) Representative histograms show changes in the PI-signal - DNA content (x-axis) versus cell number (y-axis) in cells exposed to the C-2028. (**c)** Bar graphs represent data as the median with an interquartile range. Statistical analysis was performed using the Kruskal–Wallis test for non-parametric data and Dunn’s post-hoc test. Significantly different from the control at: * *p* < 0.05, ** *p* < 0.01, *** *p* < 0.001; *n* ≥ 3

Exposure of MIA PaCa-2 cells to UAs at the IC_80_ doses resulted in complete inhibition of cell proliferation (Fig. 6a; Table 9, Fig. S13 and Table S11 in the Supplementary Information). Cell division was completely blocked after administration of C-2028 at the IC_80_ dose even after 24 h. The C-2045 and C-2053 derivatives, despite causing significant reductions (Table 9) in the number of colonies formed after 24, 72, and 120 h, achieved the same effect as the C-2028 derivative only after 168 h. UAs at the IC_50_ dose caused gradual reductions in the number of colonies formed with the incubation time, and after 168 h, their number did not exceed 5 (Fig. 6a; Table 9). MIA PaCa-2 cells were able to undergo mitosis and divide after exposure to gemcitabine, and the strongest inhibition of proliferation was observed after 72 h of incubation. Interestingly, with longer incubation times (120 and 168 h), an increase in the number of colonies formed was observed (Fig. S13 and Table S11 in the Supplementary Information).

**Table 9:** The results of statistical analysis for colony formation assay in MIA PaCa-2 cells after treatment with UAs at IC_50_ and IC_80_ doses (Fig. 6a), including a profile of H-values and p-levels of significance from Kruskal-Wallis test and Dunn’s multiple comparisons test. N– sample size; H– value of Kruskal-Wallis test; *p*– significance level; * *p* < 0.05, ** *p* < 0.01, *** *p* < 0.001

			*N*	Kruskal-Wallis test		Dunn’s post hoc test (Control vs. UA)				*N*	Kruskal-Wallis test		Dunn’s post hoc test (Control vs. UA)
				H	*p-value*	*p-value*					H	*p-value*	*p-value*
MIA PaCa-2 IC_50_	24 h	Control	4	14.3	< 0.001 ***		**MIA PaCa-2 IC**_**80**_	24 h	Control	4	14.5	< 0.001 ***	
		C-2028	3			0.002 **			C-2028	3			0.002 **
		C-2045	3			0.037 *			C-2045	3			0.181
		-2053	3			0.354			C-2053	3			0.057
	72 h	Control	5	19.0	< 0.001 ***			72 h	Control	5	20.3	< 0.001 ***	
		C-2028	4			< 0.001***			C-2028	4			0.003 **
		C-2045	4			0.007 **			C-2045	4			0.027 *
		C-2053	4			0.18			C-2053	4			0.003 **
	120 h	Control	5	18.0	< 0.001 ***			120 h	Control	5	18.0	< 0.001 ***	
		C-2028	4			0.003 **			C-2028	4			0.006 **
		C-2045	4			0.003 **			C-2045	4			0.016 *
		C-2053	4			0.1			C-2053	4			0.006 **
	168 h	Control	4	14.5	< 0.001 ***			168 h	Control	4	15.6	< 0.001 ***	
		C-2028	3			0.014 *			C-2028	3			0.019 *
		C-2045	3			0.049 *			C-2045	3			0.019 *
		C-2053	3			0.027 *			C-2053	3			0.019 *

Senescence is classically defined as irreversible arrest of the cell cycle in the G1 phase due to the response to DNA damage. A study of the impact of UAs at IC_50_ doses on MIA PaCa-2 cell cycle progression revealed a gradual increase in the sub-G1 fraction, indicating DNA degradation, but most importantly, the maintenance of the G1 fraction at high levels during the incubation time (Fig. 6b–c; Table 10, and Table S12 in the Supplementary Information). The number of cells with DNA < 2 N peaked at 168 h, with percentages of 39.5, 40.4, and 30.1% for treatments with C-2028, C-2045, and C-2053, respectively. The fraction of cells in the G1 phase prevailed after 72 h of incubation for the C-2028 and C-2045 derivatives (34.5 and 50.2%, respectively) and after 120 h for the C-2053 derivative (49.5%) (Fig. 6c; Table 10). Compared to control cells, the proportions of the cell populations in the S and G2/M phases were slightly higher after 24 h, but the percentages of both fractions decreased during incubation. The distribution of polyploid cells was at a similar level to that in the control.

**Table 10:** The results of statistical analysis for cell cycle analysis (G1 fraction) of MIA PaCa-2 cells after treatment with UAs at IC_50_ doses (Fig. 6c), including a profile of H-values and p-levels of significance from Kruskal-Wallis test and Dunn’s multiple comparisons test. N– sample size; H– value of Kruskal-Wallis test; n/a– not applicable; *p*– significance level; * *p* < 0.05, ** *p* < 0.01, *** *p* < 0.001

			*N*	Kruskal-Wallis test		Dunn’s post hoc test (Control vs. UA)
				H	*p-value*	*p-value*
MIA PaCa-2 IC_50_	24 h	Control	4	12.7	< 0.001 ***	
		C-2028	4			0.007 **
		C-2045	4			0.011 *
		C-2053	4			0.704
	72 h	Control	4	3.3	0.368	n/a
		C-2028	4			n/a
		C-2045	3			n/a
		C-2053	4			n/a
	120 h	Control	4	11.2	0.002 **	
		C-2028	5			0.333
		C-2045	4			> 0.999
		C-2053	4			0.371
	168 h	Control	4	10.7	0.002 **	
		C-2028	5			0.0441 *
		C-2045	3			0.633
		C-2053	4			> 0.999

### Western blot analysis

**Fig. 7:**
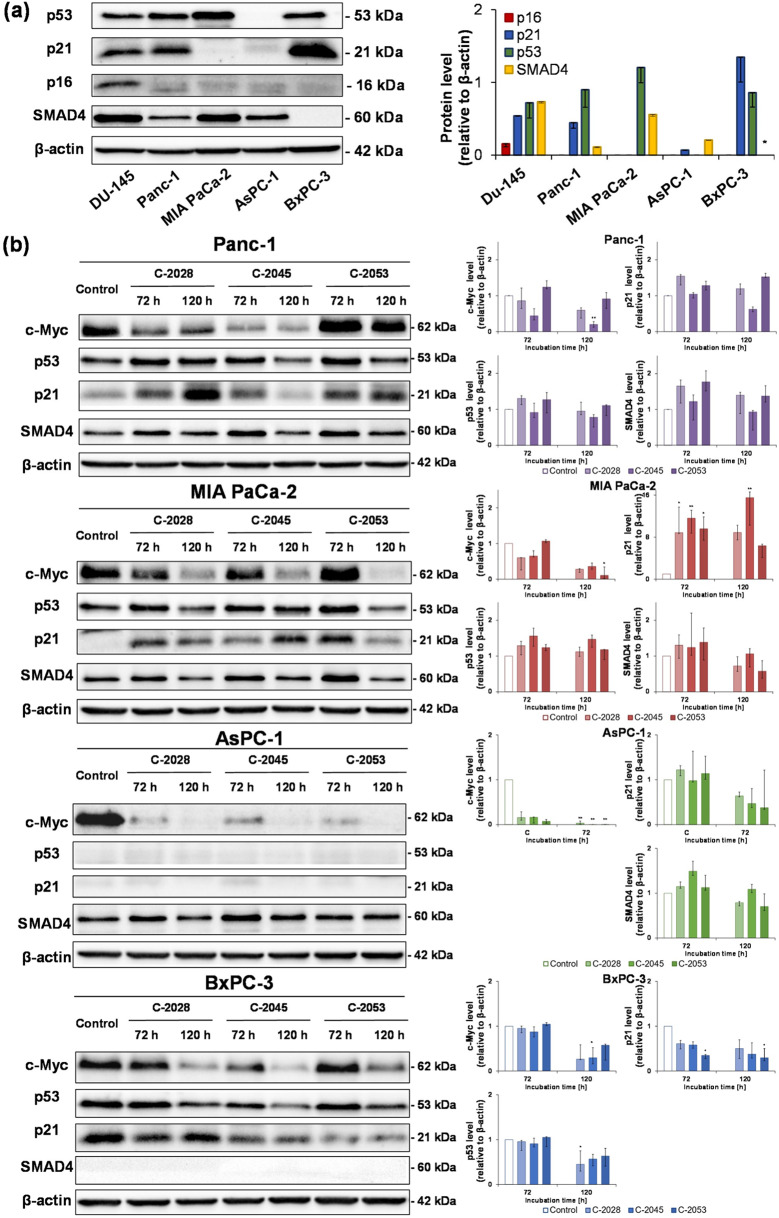
Western blot analysis of c-Myc, p53, p21, and SMAD4 protein levels. Panc-1, MIA PaCa-2, AsPC-1, and BxPC-3 cells were incubated with IC_80_ doses of C-2028, C-2045, and C-2053 for 72 and 120 h. Whole-cell extracts (30 µg of protein/lane) were prepared, separated by polyacrylamide gel electrophoresis, and transferred to the membrane by a semi-dry method. Immunoblotting and enhanced chemiluminescence (ECL) development were performed to determine protein levels by densitometric analysis. Representative Western blot analysis of (**a**) p53, p21, p16 and SMAD4 protein levels in Panc-1, MIA PaCa-2, AsPC-1 and BxPC-3 cells not treated with compounds compared to Du-145 cells serving as positive control and (**b**) c-Myc, p53, p21, and SMAD4 protein levels in Panc-1, MIA PaCa-2, AsPC-1, and BxPC-3 cells following UAs exposure. Bar graphs represent data as the median with an interquartile range. Statistical analysis was performed using the Kruskal–Wallis test for non-parametric data and Dunn’s post-hoc test. Significantly different from the control at: * *p* < 0.05, ** *p* < 0.01, *** *p* < 0.001; *n* ≥ 3

Given that a variety of mutations are observed in pancreatic cancer cells, we determined whether UAs affect the levels of proteins such as c-Myc, p53, p16 (CDKN2A), p21, and SMAD4. Moreover, we compared the levels of selected genes between the studied cell lines, as presented in Fig. 7a; Table 11. c-Myc is an oncogenic protein that plays roles in cell proliferation and the inhibition of apoptosis and, in PC, is often constitutively activated. In all four tested PC cells, significant downregulation of c-Myc was observed, especially after prolonged incubation with the three UA derivatives (Fig. 7b; Table 12). The most profound inhibition was observed in AsPC-1 cells, where even after 72 h of exposure, the protein was almost undetectable. The C-2053 compound caused the least reduction in the level of the c-Myc protein. A gene that is mutated in nearly all pancreatic cancer cell lines is *TP53*, which encodes the p53 protein. Panc-1 and MIA PaCa-2 cells expressed the p53 protein (Fig. 7a; Table 11), whose level slightly increased after 72 h of incubation and then, after 120 h, was comparable to the level of the protein in control cells (except following treatment with C-2045 in both cell lines, where the level increased in Panc-1 cells and decreased in MIA PaCa-2 cells) (Fig. 7b; Table 12). In BxPC-3 cells, the p53 protein was also detected (Fig. 7a; Table 11), and its level slightly decreased only after 120 h of incubation with all three UA derivatives (Fig. 7b; Table 12). In AsPC-1 cells, a mutation in the *TP53* gene occurred in such a region that the protein could not be created (Fig. 7a; Table 11) [29]. Western blot analysis confirmed that in these cells, the p53 protein was not detected after UAs treatment (Fig. 7b; Table 12). The level of p21 is strongly connected with p53 and the process of cell proliferation. In cells undergoing mitosis, p21 is inhibited; in turn, its level increases during cell proliferation arrest and the induction of senescence. In Panc-1 and BxPC-3 cells, p21 was observed in control untreated cells, especially in BxPC-3 cells, where the band was very thick, whereas in MIA PaCa-2 and AsPC-1 cells, p21 was not detected (Fig. 7a; Table 11). After UAs treatment in Panc-1 and MIA PaCa-2 cells, the level of p21 strongly increased, especially in MIA PaCa-2 cells, in which accelerated senescence was induced. In AsPC-1 cells, although inhibition of proliferation and apoptosis was observed, p21 was not detected upon UAs treatment (Fig. 7b; Table 12). In BxPC-3 cells, the control level of p21 was very high in comparison with the levels in the other cell lines tested (Fig. 7a; Table 11), and during incubation with UA compounds, its level decreased, especially after C-2045 and C-2053 exposure (Fig. 7b; Table 12). Notably, in these cells, cell division inhibition and apoptosis induction were observed in the smallest population of cells compared with the other cell lines. The next suppressor gene that is mutated in different types of cancer is *CDKN2A*, which encodes the p16 protein. In all four studied cell lines, p16 was not detected, indicating the deletion of this gene in pancreatic cells (Fig. 7a; Table 11). Another protein involved in pancreatic cancer initiation and progression is SMAD4, which is a crucial mediator protein in the TGF-β signaling pathway. Three studied cell lines, Panc-1, MIA PaCa-2, and AsPC-1, expressed this protein, whereas the protein was not detected in BxPC-3 cells (Fig. 7a; Table 11). In PC cells exposed to UAs for 72 h, the level of SMAD4 slightly increased, and after 120 h, it returned to the level of that in control untreated cells’ (Fig. 7b; Table 12).

**Table 11:** The results of statistical analysis for Western blot analysis of p16, p21, p53, and SMAD4 protein levels in untreated pancreatic cancer cells (PC) (Fig. 7a), including a profile of H-values and p-levels of significance from Kruskal-Wallis test and Dunn’s multiple comparisons test. N– sample size; H– value of Kruskal-Wallis test; n/a - not applicable; *p* - significance level; * *p* < 0.05, ** *p* < 0.01, *** *p* < 0.001

		*N*	Kruskal-Wallis test		Dunn’s post hoc test (Du-145 vs. PC)			*N*	Kruskal-Wallis test		Dunn’s post hoc test (Du-145 vs. PC)
			H	*p-value*	*p-value*				H	*p-value*	*p-value*
p16	Du-145	2	8.9	0.111		**p21**	Du-145	2	8.8	0.001 **	
	Panc-1	2			n/a		Panc-1	2			> 0.999
	MIA PaCa-2	2			n/a		MIA PaCa-2	2			0.187
	AsPC-1	2			n/a		AsPC-1	2			0.74
	BxPC-3	2			n/a		BxPC-3	2			> 0.999
p53	Du-145	2	6.1	0.173		**SMAD4**	Du-145	2	8.8	0.001 **	
	Panc-1	2			n/a		Panc-1	2			0.187
	MIA PaCa-2	2			n/a		MIA PaCa-2	2			> 0.999
	AsPC-1	2			n/a		AsPC-1	2			0.74
	BxPC-3	2			n/a		BxPC-3	2			0.032 *

**Table 12:** The results of statistical analysis for Western blot analysis of c-Myc, p53, p21, and SMAD4 protein levels in pancreatic cancer cells after treatment with UAs at IC_80_ doses (Fig. 7b), including a profile of H-values and p-levels of significance from Kruskal-Wallis test and Dunn’s multiple comparisons test. N– sample size; H– value of Kruskal-Wallis test; n/a - not applicable; *p* - significance level; * *p* < 0.05, ** *p* < 0.01, *** *p* < 0.001

				*N*	Kruskal-Wallis test		Dunn’s post hoc test (Control vs. UA)					*N*	Kruskal-Wallis test		Dunn’s post hoc test (Control vs. UA)	
			[h]		H	*p-value*	*p-value*				[h]		H	*p-value*	*p-value*	
Panc-1	c-Myc	Control		4	26.3	< 0.001 ***		**MIA PaCa-2**	c-Myc	Control		4	23.7	0.003 **		
		C-2028	72	3			> 0.999			C-2028	72	3			> 0.999	
			120	4			> 0.999				120	4			0.05	
		C-2045	72	4			0.496			C-2045	72	3			> 0.999	
			120	4			0.007 **				120	4			0.355	
		C-2053	72	3			> 0.999			C-2053	72	3			> 0.999	
			120	3			> 0.999				120	3			0.018 *	
	p53	Control		3	12.6	0.127			p53	Control		3	14.4	0.072	n/a	
		C-2028	72	3			n/a			C-2028	72	3			n/a	
			120	3			n/a				120	3			n/a	
		C-2045	72	3			n/a			C-2045	72	3			n/a	
			120	3			n/a				120	3			n/a	
		C-2053	72	3			n/a			C-2053	72	3			n/a	
			120	3			n/a				120	3			n/a	
	p21	Control		4	24.2	0.002 **			p21	Control		4	25.9	0.001 **		
		C-2028	72	3			0.205			C-2028	72	3			0.040 *	
			120	3			> 0.999				120	3			0.122	
		C-2045	72	3			> 0.999			C-2045	72	4			0.007 **	
			120	3			> 0.999				120	3			0.002 **	
		C-2053	72	3			> 0.999			C-2053	72	4			0.032 *	
			120	3			0.118				120	3			> 0.999	
	SMAD4	Control		3	17.9	0.022 *			SMAD4	Control		5	24.7	0.002 **		
		C-2028	72	3			0.511			C-2028	72	4			> 0.999	
			120	3			> 0.999				120	5			> 0.999	
		C-2045	72	3			> 0.999			C-2045	72	5			> 0.999	
			120	3			> 0.999				120	5			> 0.999	
		-2053	72	3			0.069			C-2053	72	5			> 0.999	
			120	3			> 0.999				120	5			0.568	
AsPC-1	c-Myc	Control		4	27.3	< 0.001 ***		**BxPC-3**	c-Myc	Control		4	21.5	0.006 **		
		C-2028	72	4			0.776			C-2028	72	3			> 0.999	
			120	4			0.008 **				120	3			0.206	
		-2045	72	3			0.382			C-2045	72	3			> 0.999	
			120	3			0.001 **				120	4			0.040 *	
		C-2053	72	4			0.059			C-2053	72	3			> 0.999	
			120	3			0.004 **				120	3			0.46	
	p21	Control		4	25.2	0.001 **			p53	Control		4	22.3	0.004 **		
		C-2028	72	3			> 0.999			C-2028	72	3			> 0.999	
			120	3			> 0.999				120	4			0.023 *	
		C-2045	72	3			> 0.999			C-2045	72	3			> 0.999	
			120	4			> 0.999				120	3			0.085	
		C-2053	72	3			> 0.999			C-2053	72	3			> 0.999	
			120	3			> 0.999				120	4			0.072	
	SMAD4	Control		4	28.4	< 0.001 ***			p21	Control		4	23.3	0.003 **		
		C-2028	72	3			> 0.999			C-2028	72	4			> 0.999	
			120	3			> 0.999				120	4			0.516	
		C-2045	72	4			0.27			C-2045	72	4			> 0.999	
			120	3			> 0.999				120	4			0.252	
		C-2053	72	4			> 0.999			C-2053	72	4			0.029 *	
			120	4			> 0.999				120	4			0.018 *	

In the positive control cells, changes in the levels of the studied proteins were also observed (Fig. 3g–h; Table 13). After 120 h of incubation, the c-Myc levels in Panc-1 and MIA PaCa-2 cells significantly decreased (Fig. 3h; Table 13), whereas in AsPC-1 and BxPC-3 cells, only slight downregulation was triggered. Irinotecan did not affect the level of p53 in Panc-1 cells, whereas gemcitabine caused a decrease in its expression in MIA PaCa-2 and BxPC-3 cells. In all four tested cell lines treated with positive control drugs, an increase in p21 was noted, with high levels in Panc-1 and AsPC-1 cells (in UAs-treated cells, p21 was not detected) and lower levels in MIA PaCa-2 and BxPC-3 cells. The level of SMAD4 changed differently upon positive control drug treatment. Irinotecan increased the level of SMAD4 in Panc-1 cells, whereas gemcitabine decreased it in MIA PaCa-2 and AsPC-3 cells.

**Table 13:** The results of statistical analysis for Western blot analysis of c-Myc, p53, p21, and SMAD4 protein levels in pancreatic cancer cells after treatment with positive controls (+): irinotecan (IR) or gemcitabine (GEM) (Fig. 3h), including a profile of H-values and p-levels of significance from Kruskal-Wallis test and Dunn’s multiple comparisons test. N– sample size; H– value of Kruskal-Wallis test; n/a - not applicable; *p* - significance level; * *p* < 0.05, ** *p* < 0.01, *** *p* < 0.001

			Kruskal-Wallis test		Dunn’s post hoc test (Control vs. (+))				Kruskal-Wallis test		Dunn’s post hoc test (Control vs. (+))
		N	H	*p-value*	*p-value*			N	H	*p-value*	*p-value*
Panc-1	Control	4	26.3	< 0.001 ***		**MIA PaCa-2**	Control	4	23.7	0.003 **	
	c-Myc	4			0.07		c-Myc	3			0.009 **
	Control	3	12.6	0.127			Control	3	14.4	0.072	
	p53	3			n/a		p53	3			n/a
	Control	4	24.2	0.002 **			Control	4	25.9	0.001 **	
	p21	4			0.013 *		p21	3			> 0.999
	Control	3	17.9	0.022 *			Control	5	24.7	0.002 **	
	SMAD4	3			0.165		SMAD4	4			0.110
AsPC-1	Control	4	27.3	< 0.001 ***		**BxPC-3**	Control	4	21.5	0.006 **	
	c-Myc	3			> 0.999		c-Myc	3			0.022 *
	Control	4	25.2	0.001 **			Control	4	22.3	0.004 **	
	p21	4			0.285		p53	3			0.218
	Control	4	28.4	< 0.001 ***			Control	4	23.3	0.003 **	
	SMAD4	4			0.420		p21	3			> 0.999

## Discussion

In the present study, we showed that our recently patented compounds, unsymmetrical bisacridines exhibited high antiproliferative activity against the pancreatic cancer cell lines Panc-1, MIA PaCa-2, AsPC-1, and BxPC-3, which display different genetic profiles. UAs inhibited cell proliferation at very low concentrations, leading to induction of cell death via apoptosis in a p53-independent manner. Additionally, in MIA PaCa-2 cells, accelerated senescence was also observed through the upregulation of the p21 protein; however, this process seemed to be transient and ultimately led to cell death. c-Myc inhibition is involved in the induction of apoptosis.

Considering the high cytotoxic and antitumor activity of the UAs against pancreatic cancer cells [24] and the unusual mechanism of interaction of UAs with DNA [25], here we decided to examine the cellular mechanism of action of bisacridine derivatives in different pancreatic cancer cell lines and their impact on proteins that are often mutated in PC or possess G-quadruplexes in the promoter regions of their genes. Our research included the following cell lines: Panc-1, MIA PaCa-2, AsPC-1, and BxPC-3, which were treated with three different derivatives of unsymmetrical bisacridines and an appropriate positive controls. All three tested compounds exhibited very strong antiproliferative effects on the four cell lines. The IC_50_ values ranged from 0.025 µM (average dose against Panc-1) to 0.2 µM (for BxPC-3 cells treated with C-2045). The IC_80_ values were also very low and did not exceed the concentration of 0.5 µM. All the cell lines used in this study were very sensitive to the UA compounds, and C-2028 was the most active compound, whereas C-2045 was the least active. C-2045 possesses a hydroxyl group in its structure, and this group undergoes glucuronidation, which could explain why this derivative has the lowest activity among others [30]. In contrast, the positive control drugs had weaker antiproliferative effects on the tested cell lines. Panc-1 cells are not very sensitive to several widely used clinical medicines, such as gemcitabine, erlotinib, 5-fluorouracil, and cisplatin, for which the IC_50_ values are very high or cannot be established in the concentration range used. Irinotecan administration presented moderate growth inhibition; therefore, this compound was chosen as a positive control in studies triggered on Panc-1 cells. Gemcitabine appeared to be the best and most active clinically used compound against MIA PaCa-2, AsPC-1, and BxPC-3 cells, as the IC_50_ values were very low, comparable to those of the C-2028 derivative (in the case of AsPC-1 and BxPC-3, not for MIA PaCa-2, for which it was the weakest drug compared with UAs). However, most importantly, the IC_80_ values could not be determined for any of the cell lines. Promisingly, the lower toxicity of UAs against normal pancreatic cells of hTERT-HPNE indicates good selectivity of the tested compounds, which greatly underlines their value as potential agents against pancreatic cancer.

Regarding the cellular mechanism of action, all three unsymmetrical bisacridine derivatives induced time-dependent DNA degradation in the four PC cell lines. This process was the strongest in Panc-1 cells treated with the C-2045 derivative, in MIA PaCa-2 cells treated with C-2028, and in AsPC-1 and BxPC-3 cells treated with the C-2053 derivative. The main type of cell death triggered by UAs in the four human pancreatic cancer cell lines was apoptosis, which was confirmed by annexin V-FITC and PI staining and microscopic observation of the cell nuclei. Necrosis was induced in the PC cells at very low rates under bisacridine treatment. In Panc-1 and MIA PaCa-2 cells, the translocation of phosphatidylserine was triggered in larger populations of cells than in AsPC-1 and BxPC-3 cells. Notably, features of apoptosis in cells treated with C-2028 and C-2045 for Panc-1 cells and C-2028 for MIA PaCa-2 cells were observed much earlier than those in other cell lines and with other derivatives. Microscopic observations also revealed that the most profound induction of apoptosis was observed in Panc-1, MIA PaCa-2, and AsPC-1 cells treated with UAs, whereas this induction was much weaker in BxPC-3 cells. In addition, the exposure of the cells to UAs led to an enlargement of the cell nuclei compared with those of the control cells, except for the BxPC-3 cells.

The strong induction of apoptosis in pancreatic cancer cells by bisacridine derivatives can be explained by the inhibition of the c-Myc protein triggered by these compounds. c-Myc is often overexpressed in pancreatic cancer cells and can promote proliferation, metastasis, chemoresistance, and change metabolism and the immune system response [6]. This is the reason why molecules that can inhibit c-Myc are needed [9]. All UA compounds led to the downregulation of c-Myc in the four tested cell lines. The effect was stronger after prolonged incubation, and after 120 h, c-Myc almost disappeared in some of the UA-treated cells. Our results revealed that one of the molecular targets of UA compounds can be c-Myc, which can contribute to extensive cell death. Interestingly, the apoptosis triggered in PC cells was p53 independent. Three cell lines, Panc-1, MIA PaCa-2, and BxPC-3, express a mutant p53 protein that is unable to bind to target genes, as the DNA-binding domain is disrupted [31, 32]. In AsPC-1 cells, a mutation in the *TP53* gene leads to the complete disappearance of the p53 protein [29]. Moreover, in this cell line, the p21 protein is expressed at a very low level and is not regulated by UAs. The tested compounds in the three p53-expressing PC cell lines caused slight downregulation of this protein after prolonged incubation. The levels of p21 underwent many alternations upon UAs treatment. In Panc-1 and MIA PaCa-2 cells, significant upregulation of p21 was observed, although extensive cell death through apoptosis was observed. p21 is said to be an inhibitor of apoptosis; however, it has been shown that apoptosis can occur in cells where p21 is induced and that these two processes can stimulate each other [33]. In MIA PaCa-2 control cells, the p21 protein was not detected, but its expression increased significantly after UAs treatment, the most strongly among the cell lines tested. Interestingly, in BxPC-3 cells, the initial level of p21 was very high, and after exposure to UAs, it decreased, especially in C-2053-treated cells, where apoptosis was slightly more induced. The high level of p21 can explain why the BxPC-3 cell line is not prone to undergo apoptosis. Many studies have shown that p21 can act as an oncogenic protein and promotes tumorigenesis and cancer through the inhibition of apoptosis and cell cycle arrest and the induction of the senescence process [34, 35]. The UA compounds and the positive control drug gemcitabine were not able to induce cell death to a similar extent as observed in the other tested cell lines. The slight downregulation of p21 observed in C-2053-treated BxPC-3 cells facilitated the induction of cell death. Another unusual feature of the BxPC-3 cell line is that these cells lack the SMAD4 protein, as our data and reports in the literature have shown [36]. The SMAD4 protein plays a tumor-suppressive role, and cells lacking this protein avoid cell cycle arrest and apoptosis [37]. This finding may also explain why triggering cell death in BxPC-3 cells with drugs is difficult. In the Panc-1, MIA PaCa-2, and AsPC-1 cell lines, the SMAD4 protein was detected with the wild-type variant (for AsPC-1 cells, the results of the variant are inconsistent, although more studies point to the wild type) [31]. After 72 h of incubation, all the UA compounds upregulated SMAD4 levels in the abovementioned cell lines, which could help in the induction of apoptosis in these cells.

Another studied cellular effect induced by UAs was accelerated senescence, and microscopic observations of X-gal-stained cells revealed that this process was triggered only in MIA PaCa-2 cells, especially after C-2045 treatment. This finding was confirmed by performing a quantitative cytometric analysis of cells with active SA-β-galactosidase. Interestingly, the cells of the MIA PaCa-2 line, which presented features characteristic of senescence after the first few days, seemed to die via apoptosis after prolonged incubation. Some of the cells that were stained blue after the metabolic transformation of X-gal shrank, which is typical for apoptotic blebs that form apoptotic bodies. Senescent cells are resistant to apoptosis [38, 39]. However, microscopic observation of MIA PaCa-2 cells treated with UA compounds revealed unusual behavior, in which senescent cells were forced to undergo apoptosis. This unique observation should be investigated more thoroughly, as in the senolytic field of research, such behavior is in demand [40]. Moreover, MIA PaCa-2 cells underwent irreversible inhibition of proliferation after treatment with the IC_80_ doses of the UAs. In contrast, the IC_50_ doses of the tested bisacridines, which mainly induced accelerated cellular senescence, significantly reduced the proliferative capacity of the cells. In addition, cell cycle analysis confirmed that MIA PaCa-2 cells underwent senescence, as indicated by their accumulation in the G1 phase, and the increasing percentage of the sub-G1 cell fraction demonstrated that this dose also induced cell death. The greatest increase in p21 protein levels, up to 14 times greater than that in the controls, was observed in MIA PaCa-2 cells, confirming the induction of cellular senescence through the activation of p21. The p16 protein [41] did not participate in the induction of senescence in MIA PaCa-2 cells because none of the tested PC cell lines expressed this protein, as shown by our results and those in the literature [42, 43].

To summarize, unsymmetrical bisacridine derivatives can be revealed as potent antitumor compounds against pancreatic cancer cells. These compounds exhibit high antiproliferative activity at very low concentrations in PC cells. Although the positive control drugs irinotecan in Panc-1 cells and gemcitabine in MIA PaCa-2, AsPC-1, and BxPC-3 cells triggered effects similar to those of the UA compounds, there are significant differences that demonstrate that UAs are highly potent compounds. The most precise example of this is the BxPC-3 cell line, the only one evaluated that lacks SMAD4 expression. Our tests revealed that the most active derivative only against this cell line was C-2053, and more importantly, this compound induced apoptosis in a two-fold greater cell population than the positive control, gemcitabine. Moreover, C-2028 appeared to be very active in MIA PaCa-2 cells at particularly low concentrations. It caused cell death to the greatest extent and completely inhibited cell proliferation at the IC_80_ dose and almost completely at the IC_50_ dose. This study also revealed that one of the molecular targets of UAs might be c-Myc, which can facilitate the induction of cell death in a p53-independent manner. Apoptosis has been identified as the main mechanism of cell death, and induction of the SMAD4 protein can facilitate this process. The intensity of c-Myc inhibition may contribute to the efficacy of all UA compounds, as may the initial level of p21 protein in control cells. Moreover, UAs can also induce accelerated senescence in a dose-dependent manner through the upregulation of the p21 protein. This phenomenon has been described in several publications, where compounds administered at lower doses or shorter treatment times can induce senescence, whereas higher doses or prolonged incubation times tend to switch the response toward triggering apoptosis [44–46]. We demonstrated such a potential of UAs, as they induced senescence in a dose-dependent manner only in MIA PaCa-2 cells. Importantly, prolonging the incubation time with UAs, even at a lower dose, resulted in the appearance of an apoptotic phenotype in senescent cells. This dose-dependent switch between senescence and apoptosis induction provides an alternative therapeutic approach with reduced toxicity and the potential for synergistic combinations of UAs with immune activators [47] or senolytic agents [45].

The use of cell lines with distinct genetic profiles that more accurately represent the diversity in the patient population has revealed that UAs are widely effective, increasing the likelihood of successful translation into clinical practice. Although unsymmetrical bisacridines exhibit unique and desirable features as candidates for anticancer therapy, further research on their molecular targets and interactions with specific cellular pathways must be undertaken to fully understand the anticancer potential of these compounds.

## Electronic supplementary material

Below is the link to the electronic supplementary material.

Supplementary Material 1

## Data Availability

Data obtained during this study are included in this article and Supplementary Information file. Detailed data are available from the corresponding author upon reasonable request.

## Abbreviations

ATCC: American Type Culture Collection
Bcl-2: B-cell lymphoma 2
C-2028: 9’-{N-[(imidazo[4,5,1-de]-acridin-6-on-5-yl)aminopropyl]-N-methylaminopropylami-no}-1’-nitroacridine×1.5HCl
C-2045: 9’-{N-[(8-hydroxyimidazo[4,5,1-de]-acridin-6-on-5-yl)aminopropyl]-N-methylaminopropylamino}-4’-methyl-1’-nitroacridine×3HCl
C-2053: 9’-{N-[(imidazo[4,5,1-de]-acridin-6-on-5-yl)aminopropyl]-N-methylaminopropylamino}-4’-methyl-1’-nitroacridine×3HCl
CDKN2A: Cyclin Dependent Kinase Inhibitor 2 A
c-Myc: Myelocytomatosis Oncogene
DMEM HG: Dulbecco’s Modified Eagle’s Medium High-Glucose
DMSO: Dimethyl Sulfoxide
ECL: Enhanced Chemiluminescence
EMT: Epithelial-Mesenchymal Transition
FACS: Fluorescence-Activated Cell Sorting
FBS: Fetal Bovine Serum
FITC: Fluorescein Isothiocyanate
FOLFIRINOX: Multidrug: fOlinic Acid, Fluorouracil, Irinotecan, and Oxaliplatin
Kit: Tyrosine-Protein Kinase
K-Ras: Kirsten Rat Sarcoma Viral Oncogene Homolog
MTT: 3-(4,5-Dimethylthiazol-2-yl)-2,5-diphenyltetrazolium bromide
n: Number of Repetitions
PBS: Phosphate-Buffered Saline
PC: Pancreatic Cancer
PCNA: Proliferating Cell Nuclear Antigen
PDGFRB: Platelet-Derived Growth Factor Receptor Beta
PI: Propidium Iodide
RIPA: Radioimmunoprecipitation Assay Buffer
RPMI 1640: Roswell Park Memorial Institute Medium
SA-β-Gal: Senescence Associated β-Galactosidase
SD: Standard Deviation
SDS-PAGE: Sodium Dodecyl Sulfate-Polyacrylamide Gel Electrophoresis
SMAD4: Mothers Against Decapentaplegic Homolog 4
TBST: Tris-Buffered Saline With Tween^®^20
TGF-β: Transforming Growth Factor-β
UAs: Unsymmetrical Bisacridines
X-Gal: 5-bromo-4-chloro-3-indolyl-β-D-Galactosidase
VEGF: Vascular Endothelial Growth Factor

## References

[1] Barcellini A, Peloso A, Pugliese L, Vitolo V, Cobianchi L. Locally advanced pancreatic ductal adenocarcinoma: challenges and progress. Onco Targets Ther. 2020;13:2705–12720. 10.2147/OTT.S220971. 10.2147/OTT.S220971 PMC7737010 33335406

[2] Gheorghe G, Bungau S, Ilie M, Behl T, Vesa CM, Brisc C, et al. Early diagnosis of pancreatic Cancer: the Key for Survival. Diagnostics. 2020;10:869. 10.3390/diagnostics10110869. 33114412 10.3390/diagnostics10110869 PMC7694042

[3] Gutiérrez ML, Muñoz-Bellvís L, Orfao A. Genomic heterogeneity of pancreatic ductal adenocarcinoma and its clinical impact. Cancers. 2021;13:4451. 10.3390/cancers13174451. 34503261 10.3390/cancers13174451 PMC8430663

[4] Singh D, Upadhyay G, Srivastava RK, Shankar S. Recent advances in pancreatic cancer: biology, treatment, and prevention. Biochim Biophys Acta. 2015;1856:13–27. 10.1016/j.bbcan.2015.04.003. 25977074 10.1016/j.bbcan.2015.04.003

[5] Wood LD, Hruban RH. Genomic landscapes of pancreatic neoplasia. J Pathol Transl Med. 2015;49:13–22. 10.4132/jptm.2014.12.26. 25812653 10.4132/jptm.2014.12.26 PMC4357405

[6] Ala M. Target c-Myc to treat pancreatic cancer. Cancer Biol Ther. 2022;23:34–50. 10.1080/15384047.2021.2017223. 34978469 10.1080/15384047.2021.2017223 PMC8812741

[7] Parasido E, Avetian GS, Naeem A, Graham G, Pishvaian M, Glasgow E, et al. The sustained induction of c-MYC drives nab-Paclitaxel Resistance in primary pancreatic ductal carcinoma cells. Mol Cancer Res. 2019;17:1815–27. 10.1158/1541-7786.MCR-19-0191. 31164413 10.1158/1541-7786.MCR-19-0191 PMC6726538

[8] Dang CV, O’Donnell KA, Zeller KI, Nguyen T, Osthus RC, Li F. The c-Myc target gene network. Semin Cancer Biol. 2006;16:253–64. 10.1016/j.semcancer.2006.07.014. 16904903 10.1016/j.semcancer.2006.07.014

[9] Wirth M, Mahboobi S, Krämer OH, Schneider G. Concepts to target MYC in pancreatic Cancer. Mol Cancer Ther. 2016;15:1792–8. 10.1158/1535-7163.MCT-16-0050. 27406986 10.1158/1535-7163.MCT-16-0050

[10] Kreis NN, Louwen F, Yuan J. The multifaceted p21 (Cip1/Waf1/CDKN1A) in cell differentiation, Migration and Cancer Therapy. Cancers (Basel). 2019;11:1220. 10.3390/cancers11091220. 31438587 10.3390/cancers11091220 PMC6770903

[11] Kitaura H, Shinshi M, Uchikoshi Y, Ono T, Iguchi-Ariga SM, Ariga H. Reciprocal regulation via protein-protein interaction between c-Myc and p21(cip1/waf1/sdi1) in DNA replication and transcription. J Biol Chem. 2000;275:10477–83. 10.1074/jbc.275.14.10477. 10744738 10.1074/jbc.275.14.10477

[12] Warfel NA, El-Deiry WS. p21WAF1 and tumourigenesis: 20 years after. Curr Opin Oncol. 2013;25:52–8. 10.1097/CCO.0b013e32835b639e. 23159848 10.1097/CCO.0b013e32835b639e

[13] Mizrahi JD, Surana R, Valle JW, Shroff RT. Pancreatic cancer. Lancet. 2020;395:2008–20. 10.1016/S0140-6736(20)30974-0. 32593337 10.1016/S0140-6736(20)30974-0

[14] Morton JP, Timpson P, Karim SA, Ridgway RA, Athineos D, Doyle B, et al. Mutant p53 drives metastasis and overcomes growth arrest/senescence in pancreatic cancer. Proc Natl Acad Sci U S A. 2010;107:246–51. 10.1073/pnas.0908428107. 20018721 10.1073/pnas.0908428107 PMC2806749

[15] Cortesi M, Zanoni M, Pirini F, Tumedei MM, Ravaioli S, Rapposelli IG, et al. Pancreatic Cancer and Cellular Senescence: Tumor Microenvironment under the spotlight. Int J Mol Sci. 2021;23:254. 10.3390/ijms23010254. 35008679 10.3390/ijms23010254 PMC8745092

[16] Racu ML, Lebrun L, Schiavo AA, Van Campenhout C, De Clercq S, Absil L, et al. The role of SMAD4 inactivation in epithelial-mesenchymal plasticity of pancreatic ductal adenocarcinoma: the missing link? Cancers. 2022;14:973. 10.3390/cancers14040973. 35205719 10.3390/cancers14040973 PMC8870198

[17] Conroy T, Desseigne F, Ychou M, Bouché O, Guimbaud R, Bécouarn Y, et al. FOLFIRINOX versus gemcitabine for metastatic pancreatic cancer. N Engl J Med. 2011;364:1817–25. 10.1056/NEJMoa1011923. 21561347 10.1056/NEJMoa1011923

[18] Von Hoff DD, Ervin T, Arena FP, Chiorean EG, Infante J, Moore M, et al. Increased survival in pancreatic cancer with nab-paclitaxel plus gemcitabine. N Engl J Med. 2013;369:1691–703. 10.1056/NEJMoa1304369. 24131140 10.1056/NEJMoa1304369 PMC4631139

[19] Neoptolemos JP, Kleeff J, Michl P, Costello E, Greenhalf W, Palmer DH. Therapeutic developments in pancreatic cancer: current and future perspectives. Nat Rev Gastroenterol Hepatol. 2018;15:333–48. 10.1038/s41575-018-0005-x. 29717230 10.1038/s41575-018-0005-x

[20] Konopa J, Horowska B, Paluszkiewicz EM, Borowa-Mazgaj B, Augustin E, Skwarska A et al. October. Asymmetric bis-acridines with antitumor activity and use thereof. Eur Patent EP 3070078 B1, 4 2017.

[21] Konopa J, Horowska B, Paluszkiewicz EM, Borowa-Mazgaj B, Augustin E, Skwarska A et al. Asymmetric bis-acridines with antitumour activity and their uses. U S Patent US10,202,349 B2, 12 February 2019.

[22] Konopa J, Horowska B, Paluszkiewicz EM, Borowa-Mazgaj B, Augustin E, Skwarska A, et al. Asymmetric bis-acridines with antitumour activity and their uses. Japanese Patent no. February 2023;7226918:13.

[23] Konopa J, Horowska B, Paluszkiewicz EM, Borowa-Mazgaj B, Augustin E, Skwarska A et al. Asymmetric bis-acridines with antitumour activity and their uses. Can Patent No 2 980 084, 07 November 2023.

[24] Paluszkiewicz E, Horowska B, Borowa-Mazgaj B, Peszyńska-Sularz G, Paradziej-Łukowicz J, Augustin E, et al. Design, synthesis and high antitumor potential of new unsymmetrical bisacridine derivatives towards human solid tumors, specifically pancreatic cancers and their unique ability to stabilize DNA G-quadruplexes. Eur J Med Chem. 2020;204:112599. 10.1016/j.ejmech.2020.112599. 32736230 10.1016/j.ejmech.2020.112599

[25] Laskowski T, Kosno M, Andrałojć W, Frackowiak JE, Borzyszkowska-Bukowska J, Szczeblewski P, et al. The interactions of monomeric acridines and unsymmetrical bisacridines (UAs) with DNA duplexes: an insight provided by NMR and MD studies. Sci Rep. 2023;13:3431. 10.1038/s41598-023-30587-y. 36859494 10.1038/s41598-023-30587-y PMC9977845

[26] Pawłowska M, Kulesza J, Augustin E. c-Myc protein level affected by Unsymmetrical Bisacridines Influences Apoptosis and Senescence Induced in HCT116 Colorectal and H460 Lung Cancer cells. Int J Mol Sci. 2022;23:3061. 10.3390/ijms23063061. 35328482 10.3390/ijms23063061 PMC8955938

[27] Bidzinska J, Cimino-Reale G, Zaffaroni N, Folini M. G-quadruplex structures in the human genome as novel therapeutic targets. Molecules. 2013;18:12368–95. 10.3390/molecules181012368. 24108400 10.3390/molecules181012368 PMC6270421

[28] Chen L, Dickerhoff J, Sakai S, Yang D. DNA G-Quadruplex in human telomeres and Oncogene promoters: structures, functions, and small molecule targeting. Acc Chem Res. 2022;55:2628–46. 10.1021/acs.accounts.2c00337. 36054116 10.1021/acs.accounts.2c00337 PMC9937053

[29] Sugimoto H, Nakamura M, Yoda H, Hiraoka K, Shinohara K, Sang M, et al. Silencing of RUNX2 enhances gemcitabine sensitivity of p53-deficient human pancreatic cancer AsPC-1 cells through the stimulation of TAp63-mediated cell death. Cell Death Discov. 2015;1:15010. 10.1038/cddiscovery.2015.10. 27551445 10.1038/cddiscovery.2015.10 PMC4981025

[30] Mieszkowska A, Nowicka AM, Kowalczyk A, Potęga A, Pawłowska M, Kosno M, et al. Metabolic Profiles of New Unsymmetrical Bisacridine Antitumor Agents in Electrochemical and Enzymatic Noncellular systems and in Tumor cells. Pharmaceuticals (Basel). 2021;14:317. 10.3390/ph14040317. 33915981 10.3390/ph14040317 PMC8066102

[31] Deer EL, González-Hernández J, Coursen JD, Shea JE, Ngatia J, Scaife CL, et al. Phenotype and genotype of pancreatic cancer cell lines. Pancreas. 2010;39:425–35. 10.1097/MPA.0b013e3181c15963. 20418756 10.1097/MPA.0b013e3181c15963 PMC2860631

[32] Ozaki T, Nakagawara A. Role of p53 in cell death and human cancers. Cancers (Basel). 2011;3:994–1013. 10.3390/cancers3010994. 24212651 10.3390/cancers3010994 PMC3756401

[33] Abbas T, Dutta A. p21 in cancer: intricate networks and multiple activities. Nat Rev Cancer. 2009;9:400– 14. 10.1038/nrc2657. PMID: 19440234. 10.1038/nrc2657 PMC2722839 19440234

[34] Karimian A, Ahmadi Y, Yousefi B. Multiple functions of p21 in cell cycle, apoptosis and transcriptional regulation after DNA damage. DNA Repair (Amst). 2016;42:63–71. 10.1016/j.dnarep.2016.04.008. 27156098 10.1016/j.dnarep.2016.04.008

[35] Muñoz-Espín D, Serrano M. Cellular senescence: from physiology to pathology. Nat Rev Mol Cell Biol. 2014;15:482–96. 10.1038/nrm3823. 24954210 10.1038/nrm3823

[36] Giehl K, Seidel B, Gierschik P, Adler G, Menke A. TGFβ1 represses proliferation of pancreatic carcinoma cells which correlates with Smad4-independent inhibition of ERK activation. Oncogene. 2000;19:4531–41. 10.1038/sj.onc.1203806. 11002426 10.1038/sj.onc.1203806

[37] Zhao M, Mishra L, Deng CX. The role of TGF-β/SMAD4 signaling in cancer. Int J Biol Sci. 2018;14:111–23. 10.7150/ijbs.23230. 29483830 10.7150/ijbs.23230 PMC5821033

[38] Lee S, Lee JS. Cellular senescence: a promising strategy for cancer therapy. BMB Rep. 2019;52:35–41. 10.5483/BMBRep.2019.52.1.294. 30526771 10.5483/BMBRep.2019.52.1.294 PMC6386234

[39] Salminen A, Ojala J, Kaarniranta K. Apoptosis and aging: increased resistance to apoptosis enhances the aging process. Cell Mol Life Sci. 2011;68:1021–31. 10.1007/s00018-010-0597-y. 21116678 10.1007/s00018-010-0597-y PMC11114781

[40] Hu L, Li H, Zi M, Li W, Liu J, Yang Y, et al. Why senescent cells are resistant to apoptosis: an insight for Senolytic Development. Front Cell Dev Biol. 2022;10:822816. 10.3389/fcell.2022.822816. 35252191 10.3389/fcell.2022.822816 PMC8890612

[41] Rayess H, Wang MB, Srivatsan ES. Cellular senescence and tumor suppressor gene p16. Int J Cancer. 2012;130:1715–25. 10.1002/ijc.27316. 22025288 10.1002/ijc.27316 PMC3288293

[42] Sun C, Yamato T, Furukawa T, Ohnishi Y, Kijima H, Horii A. Characterization of the mutations of the K-ras, p53, p16, and SMAD4 genes in 15 human pancreatic cancer cell lines. Oncol Rep. 2001;8:89–92. 10.3892/or.8.1.89. 11115575 10.3892/or.8.1.89

[43] Ghaneh P, Greenhalf W, Humphreys M, Wilson D, Zumstein L, Lemoine NR, et al. Adenovirus-mediated transfer of p53 and p16(INK4a) results in pancreatic cancer regression in vitro and in vivo. Gene Ther. 2001;8:199–208. 10.1038/sj.gt.3301394. 11313791 10.1038/sj.gt.3301394

[44] Jo H, Shim K, Jeoung D. The potential of Senescence as a target for developing Anticancer Therapy. Int J Mol Sci. 2023;24:3436. 10.3390/ijms24043436. 36834846 10.3390/ijms24043436 PMC9961771

[45] Wang L, Lankhorst L, Bernards R. Exploiting senescence for the treatment of cancer. Nat Rev Cancer. 2022;22:340–55. 10.1038/s41568-022-00450-9. 35241831 10.1038/s41568-022-00450-9

[46] Petrova NV, Velichko AK, Razin SV, Kantidze OL. Small molecule compounds that induce cellular senescence. Aging Cell. 2016;15:999–1017. 10.1111/acel.12518. 27628712 10.1111/acel.12518 PMC6398529

[47] Qin S, Schulte BA, Wang GY. Role of senescence induction in cancer treatment. World J Clin Oncol. 2018;9:180–7. 10.5306/wjco.v9.i8.180. 30622926 10.5306/wjco.v9.i8.180 PMC6314866

[48] Brochure ATCC. *Pancreatic cancer p53 hotspot mutation cell panel*. https://www.atcc.org/-/media/product-assets/documents/panels/cell-biology/pancreatic-p53.pdf?rev=5335112581f94ef6a5441e7cf47e1c97

