# Supplementary material for: c-Myc inhibition and p21 modulation contribute to unsymmetrical bisacridines-induced apoptosis and senescence in pancreatic cancer cells
Source: Pharmacol Rep. 2024 Oct 3;77(1):182–209. doi: 10.1007/s43440-024-00658-6 (PMC11743403; doi:10.1007/s43440-024-00658-6)
Supplement: Supplementary file 1 — Supplementary Material 1 [file 43440_2024_658_MOESM1_ESM.docx]

**Supplementary Information**

**c-Myc inhibition and p21 modulation contribute to unsymmetrical bisacridines-induced apoptosis and senescence in pancreatic cancer cells.**

**Agnieszka Kurdyn^1^, Monika Pawłowska^1^, Ewa Paluszkiewicz^1^, Mirosława Cichorek^2^, Ewa Augustin^1^***

^1^ Department of Pharmaceutical Technology and Biochemistry, Faculty of Chemistry, Gdańsk University of Technology, Gabriela Narutowicza Str. 11/12, 80-233 Gdańsk, Poland

^2^ Department of Embryology, Medical University of Gdańsk, Dębinki Str. 1, 80-211 Gdańsk, Poland

*Corresponding author: Ewa Augustin, Department of Pharmaceutical Technology and Biochemistry, Faculty of Chemistry, Gdańsk University of Technology, Gabriela Narutowicza Str. 11/12, 80-233 Gdańsk, Poland; e-mail: [ewa.augustin@pg.edu.pl](mailto:ewa.augustin@pg.edu.pl)

**Fig. S1**

**Fig. S1** Growth inhibition curves of Panc-1 cells after exposure to UAs compounds and positive control drugs.

**Fig. S2**

**Fig. S2** Growth inhibition curves of MIA PaCa-2 cells after exposure to UAs compounds and positive control drug.

**Fig. S3**

**Fig. S3** Growth inhibition curves of AsPC-1 cells after exposure to UAs compounds and positive control drug.

**Fig. S4**

**Fig. S4** Growth inhibition curves of BcPC-3 cells after exposure to UAs compounds and positive control drug.

**Table S1.** Cytotoxicity of UAs against hTERT-HPNE cells after 72 h of treatment. (n=4)

**Table S2.** Cell cycle distribution of Panc-1 cells after treatment with C-2028, C-2045, C-2053 (at IC_80_ dose), and irinotecan (at IC_50_ dose); n≥3.

**Table S3**. Cell cycle distribution of MIA PaCa-2 cells after treatment with C-2028, C-2045, C-2053 (at IC_80_ dose), and gemcitabine (at IC_50_ dose); n≥3.

**Table S4.** Cell cycle distribution of AsPC-1 cells after treatment with C-2028, C-2045, C-2053 (at IC_80_ dose), and gemcitabine (at IC_50_ dose); n≥3.

**Table S5.** Cell cycle distribution of BxPC-3 cells after treatment with C-2028, C-2045, C-2053 (at IC_80_ dose), and gemcitabine (at IC_50_ dose); n≥3.

**Table S6.** Analysis of asymmetry and permeability of cell membrane in Panc-1 cells after treatment with C-2028, C-2045, C-2053 (at IC_80_ dose), and irinotecan (at IC_50_ dose); n≥3.

**Table S7.** Analysis of asymmetry and permeability of cell membrane in MIA PaCa-2 cells after treatment with C-2028, C-2045, C-2053 (at IC_80_ dose), and gemcitabine (at IC_50_ dose); n≥3.

**Table S8.** Analysis of asymmetry and permeability of cell membrane in AsPC-1 cells after treatment with C-2028, C-2045, C-2053 (at IC_80_ dose), and gemcitabine (at IC_50_ dose); n≥3.

**Table S9.** Analysis of asymmetry and permeability of cell membrane in BxPC-3 cells after treatment with C-2028, C-2045, C-2053 (at IC_80_ dose), and gemcitabine (at IC_50_ dose); n≥3.

**Fig. S5**

**Fig.** **S5** Changes in nuclei morphology of Panc-1 cells after treatment with IC_80_ doses of C-2028, C-2045, C-2053, and IC_50_ dose of irinotecan for 24, 72, 120, and 168 h. Cells were fixed with Carnoy's solution, stained with Hoechst 33342, and visualized under a fluorescent microscope (x400). Thin arrows point to cells undergoing apoptosis, thick arrows to mitotic catastrophe, and arrowheads to abnormal mitosis, scale bar 20 µm; n≥3. (**a**) Representative pictures of Panc-1 cells morphology exposed to UAs and irinotecan. (**b**) Enlarged fragment of the selected image.

**Fig. S6**

**Fig.** **S5** Changes in nuclei morphology of MIA PaCa-2 cells after treatment with IC_80_ doses of C-2028, C-2045, C-2053, and IC_50_ dose of gemcitabine for 24, 72, 120, and 168 h. Cells were fixed with Carnoy's solution, stained with Hoechst 33342, and visualized under a fluorescent microscope (x400). Thin arrows point to cells undergoing apoptosis, thick arrows to mitotic catastrophe, and arrowheads to abnormal mitosis, scale bar 20 µm; n≥3. (**a**) Representative pictures of Panc-1 cells morphology exposed to UAs and irinotecan. (**b**) Enlarged fragment of the selected image.

**Fig. S7**

**Fig.** **S7** Changes in nuclei morphology of AsPC-1 cells after treatment with IC_80_ doses of C-2028, C-2045, C-2053, and IC_50_ dose of gemcitabine for 24, 72, 120, and 168 h. Cells were fixed with Carnoy's solution, stained with Hoechst 33342, and visualized under a fluorescent microscope (x400). Thin arrows point to cells undergoing apoptosis, thick arrows to mitotic catastrophe, and arrowheads to abnormal mitosis, scale bar 20 µm; n≥3. (**a**) Representative pictures of Panc-1 cells morphology exposed to UAs and irinotecan. (**b**) Enlarged fragment of the selected image.

**Fig. S8**

**Fig. S8** Changes in nuclei morphology of BxPC-3 cells after treatment with IC_80_ doses of C-2028, C-2045, C-2053, and IC_50_ dose of gemcitabine for 24, 72, 120, and 168 h. Cells were fixed with Carnoy's solution, stained with Hoechst 33342, and visualized under a fluorescent microscope (x400). Thin arrows point to cells undergoing apoptosis, thick arrows to mitotic catastrophe, and arrowheads to abnormal mitosis, scale bar 20 µm; n≥3. (**a**) Representative pictures of Panc-1 cells morphology exposed to UAs and irinotecan. (**b**) Enlarged fragment of the selected image.

**Fig. S9**

**Fig.** **S9** Analysis of accelerated senescence in Panc-1 cells after C-2028, C-2045, C-2053, and irinotecan treatment at IC_50_ and/or IC_80_ doses after 72, 120, and 168 h. Briefly, cells were fixed, incubated with X-gal (substrate for β-galactosidase) staining solution, and observed under a light microscope (x200). Representative pictures are shown; scale bar 50 µm; n≥3.

**Fig.S10**

**Fig. S10** Analysis of accelerated senescence in MIA PaCa-2 cells after C-2028, C-2045, C-2053, and gemcitabine treatment at IC_50_ and/or IC_80_ doses after 72, 120, and 168 h. Briefly, cells were fixed, incubated with X-gal (substrate for β-galactosidase) staining solution, and observed under a light microscope (x200). Representative pictures are shown; scale bar 50 µm; n≥3.

**Fig.S11**

**Fig. S11** Analysis of accelerated senescence in AsPC-1 cells after C-2028, C-2045, C-2053, and gemcitabine treatment at IC_50_ and/or IC_80_ doses after 72, 120, and 168 h. Briefly, cells were fixed, incubated with X-gal (substrate for β-galactosidase) staining solution, and observed under a light microscope (x200). Representative pictures are shown; scale bar 50 µm; n≥3.

**Fig. S12**

**Fig. S12** Analysis of accelerated senescence in BxPC-3 cells after C-2028, C-2045, C-2053, and gemcitabine treatment at IC_50_ and/or IC_80_ doses after 72, 120, and 168 h. Briefly, cells were fixed, incubated with X-gal (substrate for β-galactosidase) staining solution, and observed under a light microscope (x200). Representative pictures are shown; scale bar 50 µm; n≥3.

**Table S10.** Changes of the SA-β-galactosidase activity in MIA PaCa-2 cells after 72, 120, and 168 h of incubation with IC_50_ and IC_80_ doses of UAs and IC_50_ dose of gemcitabine in comparison to untreated control cells estimated by flow cytometry method as mean fluorescence intensity (MFI); n≥3.

**Fig. S13**

**Fig.S13** Colony formation assay of MIA PaCa-2 cells exposed to IC_50_ and IC_80_ doses of C-2028, C-2045, C-2053, and IC_50_ dose of gemcitabine for 24, 72, 120, and 168 h. Briefly, cells after the treatment were seeded onto a fresh plate, post-incubated for 14 days, fixed with ethanol, stained with Giemsa dye, and counted. Representative pictures are shown; n≥3.

**Table S11**. Colony formation assay of MIA PaCa-2 cells exposed to IC_50_ and IC_80_ doses of C-2028, C-2045, C-2053, and IC_50_ dose of gemcitabine for 24, 72, 120, and 168 h, n≥3.

**Table S12.** Cell cycle distribution of MIA PaCa-2 cells after treatment with C-2028, C-2045, and C-2053 at IC_50_ dose, n≥3.

**Table S13.** Densitometric analysis of protein level of c-Myc, p53, p21, and SMD4 in pancreatic cancer cells treated with C-2028, C-2045, C-2053 (at IC_80_ dose), and irinotecan/gemcitabine (at IC_50_ dose) for 72 and 120 h, where the internal control was β-actin; n≥3.

**Fig. S14**

**Fig. S14** Original photos taken for Western blot analysis. The red box indicates the bands presented in the Fig. 7a.

**Fig. S15**

**Fig. S15** Original photos taken for Western blot analysis. The red box indicates the bands presented in Figs. 7b and 3g for Panc-1 cells. The band of p21 protein was selected according to the ATCC Brochure [48].

**Fig. S16**

**Fig. S16** Original photos taken for Western blot analysis. The red box indicates the bands presented in Figs. 7b and 3g for MIA PaCa-2 cells. The band of p21 protein was selected according to the ATCC Brochure [48].

**Fig. S17**

**Fig. S17** Original photos taken for Western blot analysis. The red box indicates the bands presented in Figs. 7b and 3g for AsPC-1 cells. The band of p21 protein was selected according to the ATCC Brochure [48].

**Fig. S18**

**Fig. S18** Original photos taken for Western blot analysis. The red box indicates the bands presented in Figs. 7b and 3g for BxPC-3 cells. The band of p21 protein was selected according to the ATCC Brochure [48].

**Fig. S19**

**Fig. S19** Ponceau staining of membranes, which were cut, immunostained for appropriate antibodies, and underwent ECL development.
